# Bioinformatics insights into the genes and pathways on severe COVID-19 pathology in patients with comorbidities

**DOI:** 10.3389/fphys.2022.1045469

**Published:** 2022-12-14

**Authors:** Abdulrahman Mujalli, Kawthar Saad Alghamdi, Khalidah Khalid Nasser, Nuha Al-Rayes, Babajan Banaganapalli, Noor Ahmad Shaik, Ramu Elango

**Affiliations:** ^1^ Department of Genetic Medicine, King Abdulaziz University, Jeddah, Saudi Arabia; ^2^ Department of Laboratory Medicine, Faculty of Applied Medical Sciences, Umm Al-Qura University, Makkah, Saudi Arabia; ^3^ Princess Al-Jawhara Center of Excellence in Research of Hereditary Disorders (PACER-HD), King Abdulaziz University, Jeddah, Saudi Arabia; ^4^ Department of Biology, Faculty of Science, University of Hafr Al Batin, Hafar Al-Batin, Saudi Arabia; ^5^ Department of Medical Laboratory Sciences, Faculty of Applied Medical Sciences, King Abdulaziz University, Jeddah, Saudi Arabia

**Keywords:** COVID-19, SARS-CoV-2, viral infections, comorbidities, cardiovascular diseases, diabetes, obesity, MERS

## Abstract

**Background:** Coronavirus disease (COVID-19) infection is known for its severe clinical pathogenesis among individuals with pre-existing comorbidities. However, the molecular basis of this observation remains elusive. Thus, this study aimed to map key genes and pathway alterations in patients with COVID-19 and comorbidities using robust systems biology approaches.

**Methods:** The publicly available genome-wide transcriptomic datasets from 120 COVID-19 patients, 281 patients suffering from different comorbidities (like cardiovascular diseases, atherosclerosis, diabetes, and obesity), and 252 patients with different infectious diseases of the lung (respiratory syncytial virus, influenza, and MERS) were studied using a range of systems biology approaches like differential gene expression, gene ontology (GO), pathway enrichment, functional similarity, mouse phenotypic analysis and drug target identification.

**Results:** By cross-mapping the differentially expressed genes (DEGs) across different datasets, we mapped 274 shared genes to severe symptoms of COVID-19 patients or with comorbidities alone. GO terms and functional pathway analysis highlighted genes in dysregulated pathways of immune response, interleukin signaling, FCGR activation, regulation of cytokines, chemokines secretion, and leukocyte migration. Using network topology parameters, phenotype associations, and functional similarity analysis with *ACE2* and *TMPRSS2*—two key receptors for this virus-we identified 17 genes with high connectivity (*CXCL10, IDO1, LEPR, MME, PTAFR, PTGS2, MAOB, PDE4B, PLA2G2A, COL5A1, ICAM1, SERPINE1, ABCB1, IL1R1, ITGAL, NCAM1 and PRKD1*) potentially contributing to the clinical severity of COVID-19 infection in patients with comorbidities. These genes are predicted to be tractable and/or with many existing approved inhibitors, modulators, and enzymes as drugs.

**Conclusion:** By systemic implementation of computational methods, this study identified potential candidate genes and pathways likely to confer disease severity in COVID-19 patients with pre-existing comorbidities. Our findings pave the way to develop targeted repurposed therapies in COVID-19 patients.

## Introduction

Severe Acute Respiratory Syndrome Coronavirus 2 (SARS-CoV-2), commonly known as coronavirus disease (COVID-19) imposed an unprecedented challenge to the global economy, lifestyle and public healthcare. COVID-19 patients, in general, show a variable spectrum of clinical phenotypes, ranging from asymptomatic to symptomatic with mild to severe acute respiratory distress syndrome (ARDS). SARS-CoV-2 virus infects all age groups, but those over 60 years of age, especially those with a history of cardiovascular disease (CVD), obesity, diabetes, or respiratory diseases, are at an increased risk of developing severe clinical complications (Goyal et al., 2020; Grasselli et al., 2020; Holman et al., 2020; Zhou et al., 2020).

CVD is strongly linked to higher SARS-CoV-1, SARS-CoV-2, and MERS infection mortality rates across many countries (Madjid et al., 2020; Zhou et al., 2020). Several studies have found that COVID-19 patients with CVD have not only severe infection-related symptoms but also myocardial damage and arrhythmia (Madjid et al., 2020; Shi et al., 2020). Interestingly, some reports also suggest the association of cardiovascular complications like thromboembolic events, myocarditis, acute coronary syndrome, and heart failure in COVID-19 patients with no prior CVD (Wu and McGoogan, 2020).

Diabetes is another comorbidity strongly associated with the severity of COVID-19 infection. It is known that people with diabetes are inclined to contract viral infections due to impaired immune responses. Further, impaired T cell function, increased release of inflammatory mediators, especially TNFα and IL-1β, and elevated levels of *ACE2* also played a crucial role in developing severe COVID-19 symptoms and predisposed to higher mortality risk (Fang et al., 2020; Fox et al., 2021). Both CVD and diabetes are strongly linked to obesity, a risk factor that determines the clinical complications in COVID-19 patients (Malik et al., 2021). Thus, all these risk factors modulate the severity of COVID-19 and drastically reduce the patient survival rate (Hernandez-Garduno, 2020).

The pro-inflammatory cytokine status compromises innate immune response, and increased expression of the *ACE2* receptor is the key molecular feature of COVID-19 and other chronic morbidity outcomes. Other molecular factors that determine the risk and severity of COVID-19 infection are frequently mentioned (Dolan et al., 2020; Facchiano et al., 2020; Singh et al., 2021). However, biological mechanisms and specific biomarkers underlying COVID-19 with associated comorbidities are poorly understood and are still being investigated. Understanding these biological mechanisms may help in developing preventive and therapeutic strategies against COVID-19 infection in patients with such comorbidities.

The unrestricted access to genome-wide transcriptome and viral genome data has accelerated the novel scientific discoveries related to COVID-19 infection and treatments. However, this exponential growth of gene expression datasets, which largely varies in experimental designs, sample sizes, array platforms, statistical methods, and analytic findings, presents both opportunities as well as challenges to biologists in identifying novel molecular drug targets. In recent years, multidimensional computational approaches have proven their robustness, efficiency, and applicability in exploring diverse large-scale biological datasets (Banaganapalli et al., 2020; Mujalli et al., 2020). These computational methods were developed using machine learning techniques and were trained on massive amounts of multi-omic (genomics, transcriptomics, proteomics, and metabolomics) datasets. Using comprehensive computational methods, our gene expression meta-analysis aims to map the unique and shared genes and pathways from transcriptomic datasets of COVID-19 patients (with severe or fatal phenotype) suffering from chronic diseases (CVD, diabetes, obesity, and lung diseases). This study may provide a novel insight into a better understanding of why COVID-19 patients with pre-existing comorbidities are more likely to develop severe clinical complications as well.

## Methods

### Datasets selection

Microarray transcriptome data from 120 COVID-19 (critical, severe, and deceased status) and from 281 patients with chronic morbidities (153 CVD, 64 atherosclerosis, 33 diabetes, and 31 obesity) were extracted from the Gene Expression Omnibus (GEO) (https://www.ncbi.nlm.nih.gov/geo/) and China National GeneBank databases (https://db.cngb.org/). Datasets from other lung viral infections such as respiratory syncytial virus (RSV, 145), influenza (95), and MERS (12) were also compared with COVID-19 in order to reduce the background noise signals by excluding common viral infection biomarkers. The full details of biological specimens, RNA isolations, hybridizations, and data analysis methods are mentioned in their corresponding publications. The full details of the gene expression datasets used in this study are shown in Supplementary Table S1.

### Raw CEL files processing, normalization, filtration and differentially expressed genes identification

Each gene expression dataset initially underwent preprocessing, normalization, and filtration separately using the robust multiarray average algorithm in R package software version 4; (http://www.R-project.org/). The robust multiarray average (RMA) in R-affy and Lumi packages, exclusively built for the Illumina microarray platform, were used to perform background data correction and normalization. The RMA technique was chosen over other packages due to its power to detect little differential change, stable variance on a log scale, and minimize false-positive results. Then the limma package was used to select DEGs and paired t-test to analyze the statistical significance of the observed DEG patterns. The Benjamini–Hochberg correction method was used for *p*-value adjustment to the false discovery rate (FDR). The cutoff point for DEG selection was set at FDR 0.05 and |log2FC| > 1.5. The non-variant probes, the housekeeping probes, and those that are not assigned to any gene were filtered out, leaving known curated gene sets with the variant probes for further analysis.

### Identification of differentially expressed genes which are unique and/or shared across different categories of the diseases

To identify both unique and shared genes in comorbidities and COVID-19, all DEGs, including upregulated and downregulated genes, were assigned to their respective category. Shared genes were then identified using the VENN tool, available in VENNY 2.1 (https://bioinfogp.cnb.csic.es/tools/venny/). At first, the “unique DEGs for comorbidities group” were identified by intersecting genes between comorbidities and respiratory viral infections like RSV, MERS, etc. Then, the “unique DEGs to COVID-19” were identified by intersecting genes between COVID-19 and other viral lung infections. Finally, unique DEGs from both groups were compared against each other to identify shared genes between the comorbidities set and COVID-19 sets (Banaganapalli et al., 2021).

### Functional analysis of category-specific differentially expressed genes (DEGs)

To explore the pathways shared between DEGs, all the genes were first grouped into three categories: COVID-19, chronic comorbidities, and lung infections. Further Enriched Clustering Ontology analysis of each DEG group was performed using the web-based tool Metascape (https://metascape.org/).

### Cell/tissue-specific expression of the identified genes

In order to determine cell and tissue distribution of the identified genes and also to generate visualization summaries of gene functions, all the query gene lists were uploaded to three different online web tools. ARCHS4 web-tool resource in EnrichR (http://amp.pharm.mssm.edu/Enrichr) contains most published RNA-seq data from humans and mice. TSEA web tool (http://genetics.wustl.edu/jdlab/tsea/) uses RNA-seq data from 45 tissues collected from 189 individuals, and the PaGenBase database in Metascape (http://metascape.org) combines gene expression patterns from literature and data mining.

### Target genes prediction and *ACE2*/TMPRSS2 similarity analysis

The Open Target Platform is an online portal (https://www.opentargets.org/) that was used to retrieve associated drug targets. This platform contains information about the pharmacological targets of drugs used in clinical practice. Measuring gene functional similarity is an essential tool for gene clustering, function prediction, and disease identification. We performed the correlation between gene expression and gene functional similarity of the query genes as a group with *ACE2* and *TMPRSS2* using the open-source tool eXploring-Genomic Relations for enhanced interpretation web tool (XGR) (http://galahad.well.ox.ac.uk:3040/). We considered the best-match score of 0.5 or above for selecting the gene-drug association.

### Causal signaling network analysis

The interaction network of genes associated with *ACE2* and *TMPRSS2* was built using the SIGnaling Network Open Resources (SIGNOR 2.0) (http://signor.uniroma2.it). The SIGNOR 3.0 web-based tool can be used to predict activation or inactivation, connections, and interactions between biomolecules and signaling molecules. All interactions with a relaxed layout and score of “0.3–0.7″ were selected.

### Mouse phenotype enrichment analysis

The mouse phenotype model is an impactful resource for prioritizing key candidate genes involved in disease processes. In this study, we used the Mammalian Phenotype (MP) enrichment analysis in ToppGene (https://toppgene.cchmc.org/) to determine the associated phenotype of genes. Mouse phenotype characteristics associated with query gene knockout were acquired from the Mouse Genome informatics database (MGI) (http://www.informatics.jax.org/).

## Results

### Data preprocessing and differentially expressed genes identification

**FIGURE 1:**
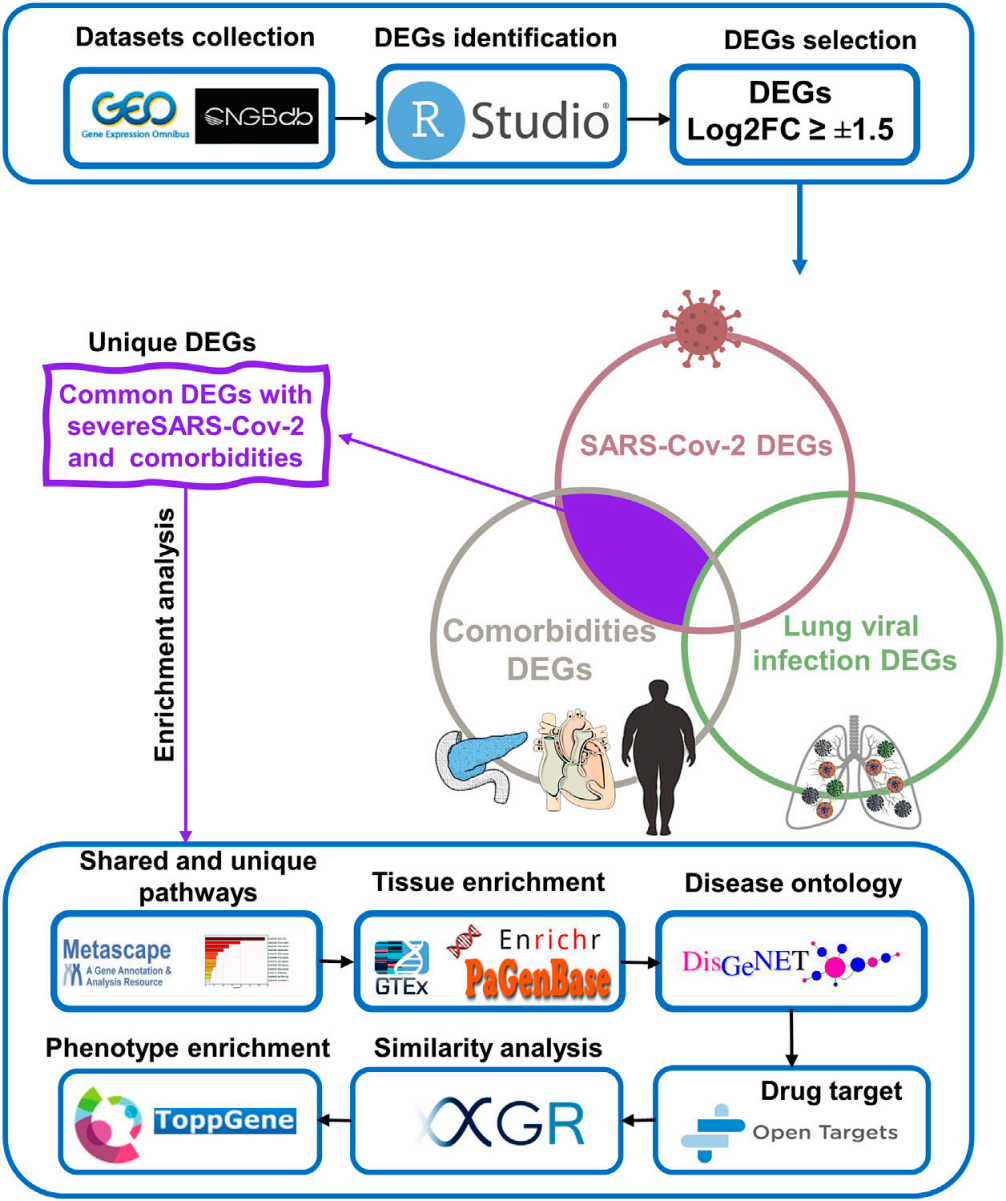
Schematic workflow of the study.

**FIGURE 2:**
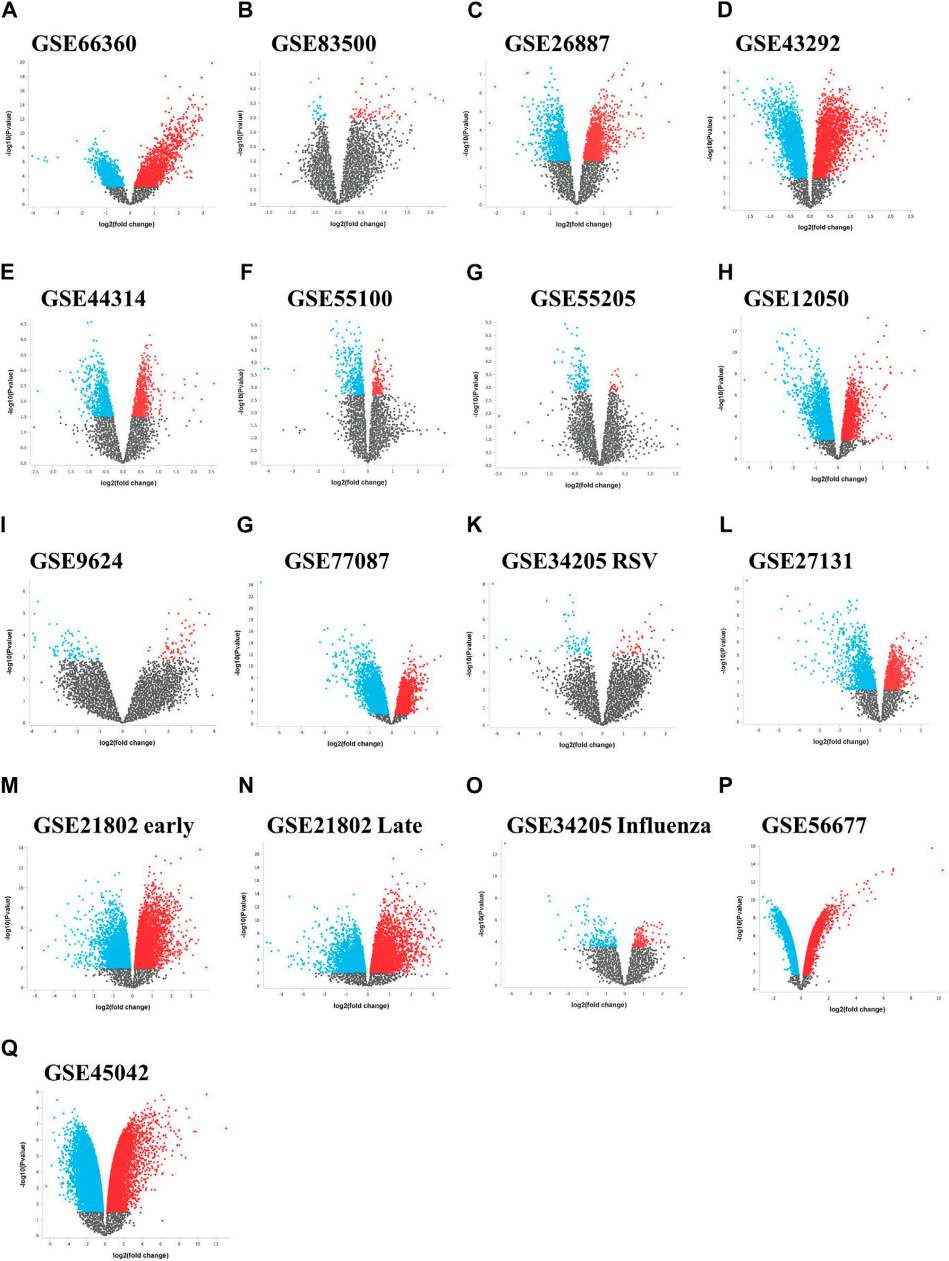
Differential expression volcano plots across data sets. Volcano plots of the gene expression data from **(A–I)** the comorbidities category, **(G–M)** lung infection category including influenza and **(P,Q)** MERS infection category. The horizontal axis represents the log2 (fold change) and the vertical axis represents the-log10 (*p*-value). The colored dots represent the significant DEGs with adjusted *p*-values < 0.05. DEGs, differentially expressed genes.

**FIGURE 3:**
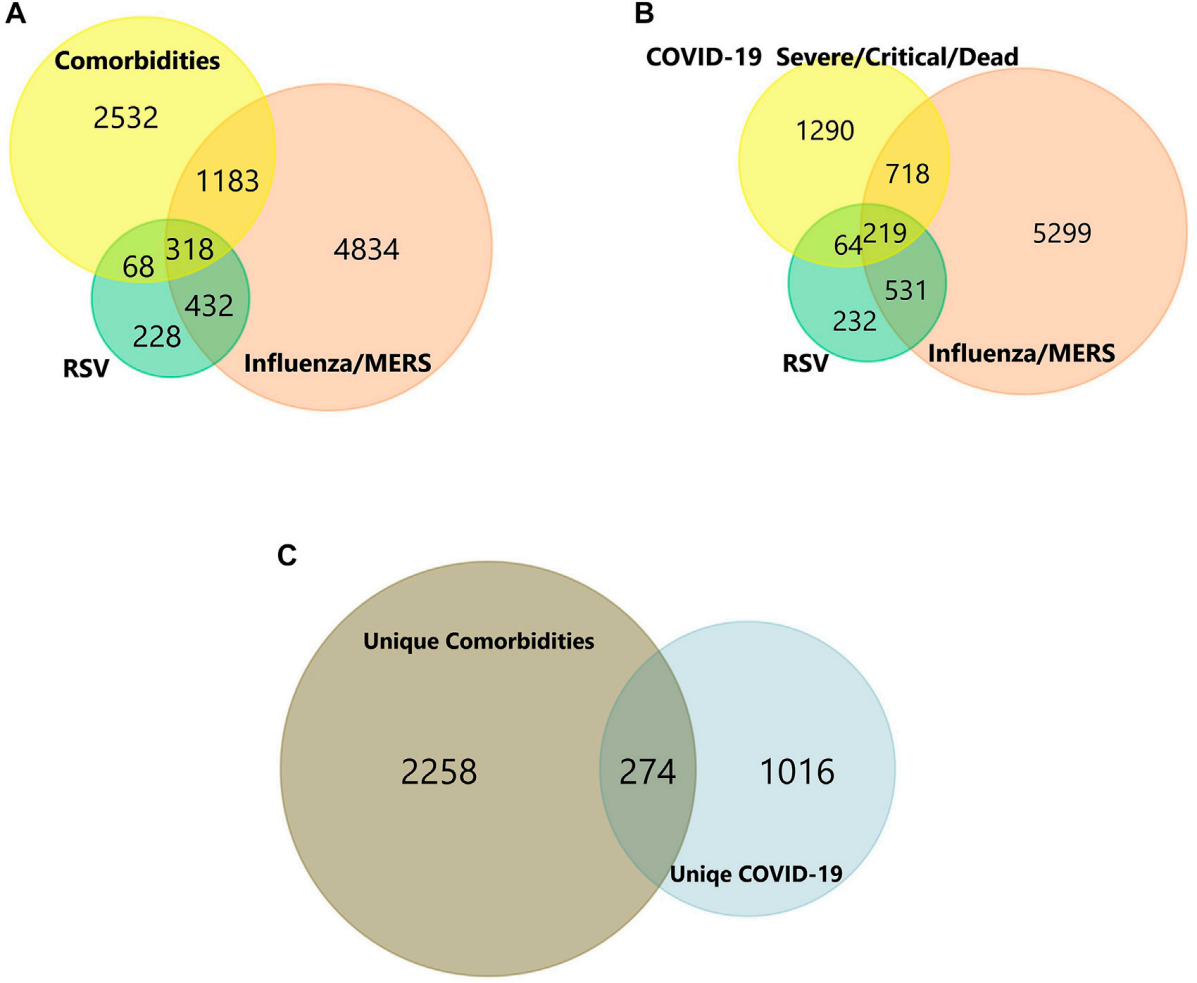
**(A-C)** Venn diagram of shared and unique genes of Comorbidities and COVID-19 infection. Colors represent different datasets. The overlapping areas are the shared DEGs. Statistically significant DEGs were defined with *p* < 0.05 and [log2FC] > 1.5 as the cut-off criterion.

We initially collected gene expression datasets from COVID-19, comorbidities (CVD, atherosclerosis, diabetes, and obesity), and lung infection (RSV, influenza, and MERS) patients (Supplementary Table S1). Each dataset was preprocessed separately using appropriate R packages and algorithm implemented in the R program and the differences in the gene expression profiles between patients and healthy participants were evaluated. Figure 1 illustrates an overview of our experimental workflow. DEGs were identified and ranked for each dataset based on their log2FC and volcano plots across all processed datasets were generated (Figure 2). For COVID-19 datasets, only DEGs of severe, critical and critical and deceased patients against the healthy individuals were selected. Subsequently, upregulated and downregulated genes were then grouped and added to their respective category, as shown in Figure 3.

### Top shared differentially expressed genes across COVID-19 and comorbidities datasets

**FIGURE 4:**
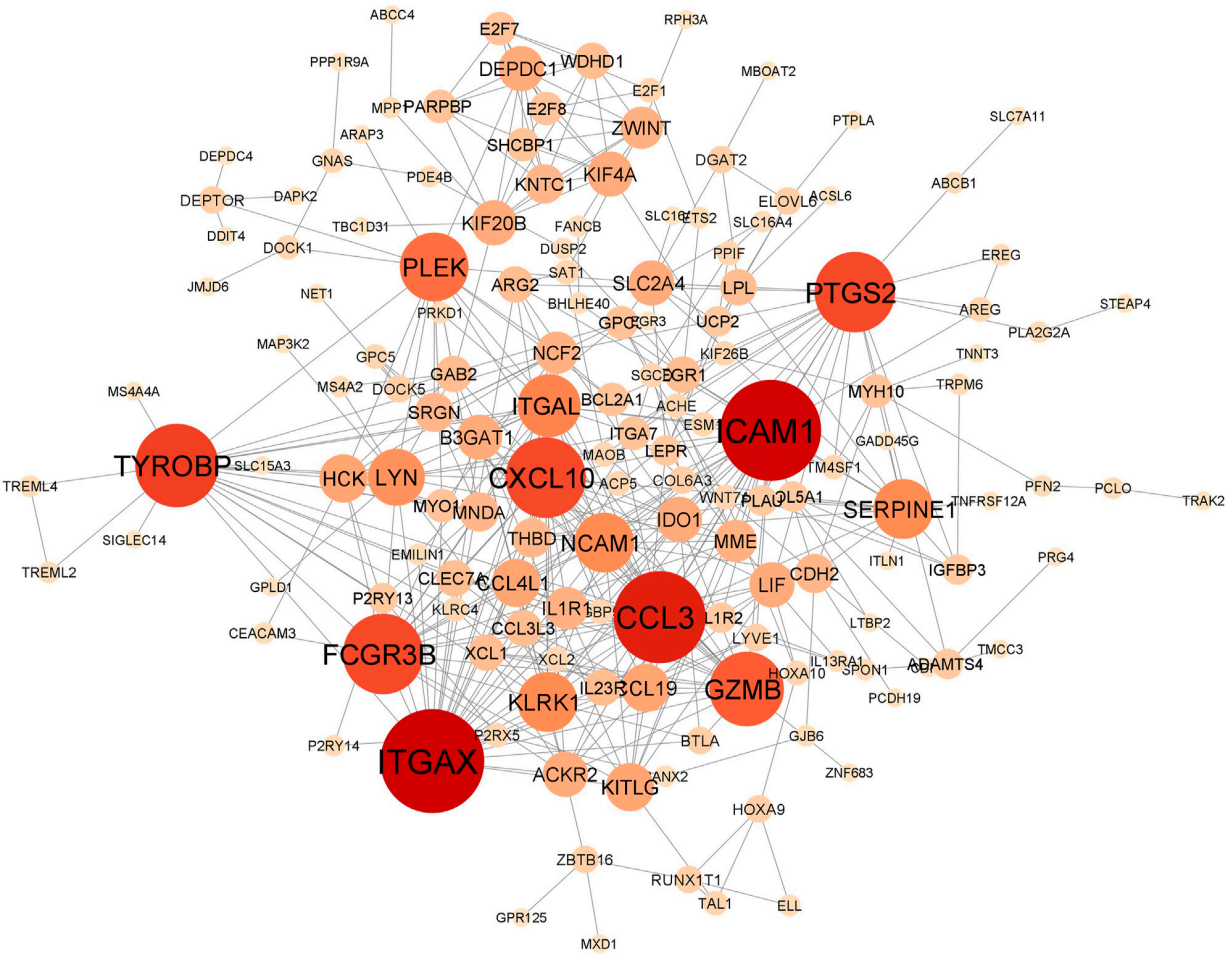
Protein-protein interaction network of 274 genes shared by the comorbidities and COVID-19 infections and classified by their degree of centrality. Node size is proportional to the degree of connection. Top gene, with at least 15 connections, includes *ITGAX, ICAM1, CCL3, TYROB, PFCGR3B, PTGS2, CXCL10, GZMB, PLEK, ITGAL, NCAM1, KLRK1* and *SERPINE1*.

In total, the comorbidities category independently contains 4,092 significantly altered genes; 10,005 in the lung infection categories, of which 4,641 and 2,291 genes were mapped from MERS and COVID-19 datasets, respectively. To identify the genes that are unique to COVID-19 pathogenesis, we mapped the common genes between COVID-19 vs. comorbidities category and eliminated them. We initially compared other lung infection datasets to comorbidities category as well. The shared DEGs were excluded and unique to comorbidities DEGs were retained for further analysis. Subsequently, the COVID-19 dataset was also compared to the lung infection category and COVID-19 unique DEGs were selected for further analysis. Our analysis showed that 2,532 genes were unique to comorbidities and 1,290 genes were unique to COVID-19 (Figures 3A,B). These unique DEGs were intersected to identify 274 genes shared between comorbidities and severe COVID-19 datasets, suggesting their potential role in the clinical severity of COVID-19 (Figure 3C) with comorbidities. Protein-Protein Interaction (PPI) network of these genes was generated with Cytoscape 3.9.1 (Figure 4). Based on the computation of a comprehensive set of topological parameters and centrality measures with the Cytoscape plugin NetworkAnalyzer, we identified 13 top-ranked genes: *ITGAX*, *ICAM1*, *CCL3*, *TYROB*, *PFCGR3B*, *PTGS2*, *CXCL10*, *GZMB*, *PLEK*, *ITGAL*, *NCAM1*, *KLRK1*, and *SERPINE1* (Figure 4).

### Cell and tissue-specific distribution of the identified genes

**FIGURE 5:**
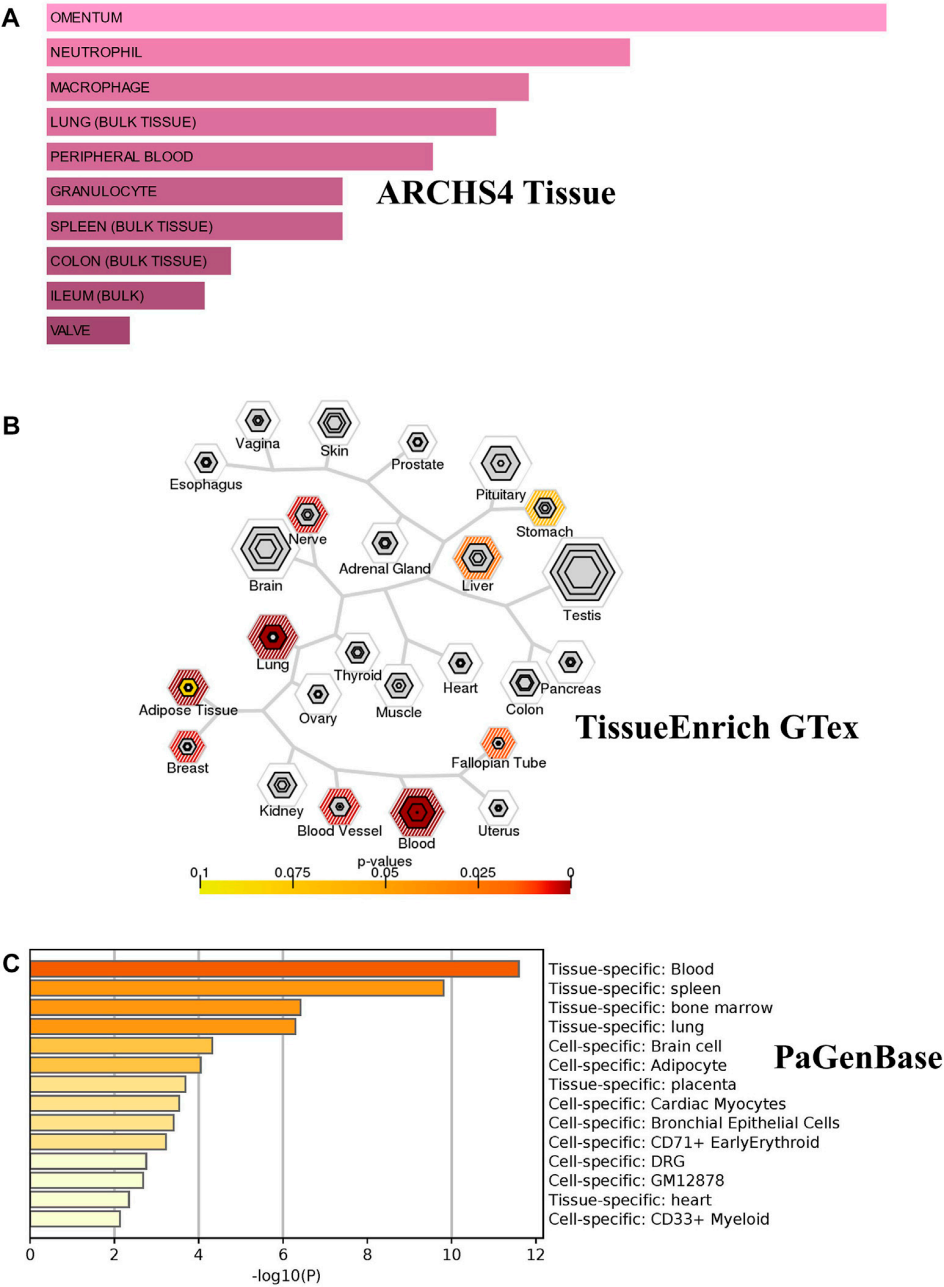
Tissue/cells-specific enrichment of Shared comorbidities-COVID-19 genes. **(A)** Enrichr Bar Graph data were collected from Enricher web (https://amp.pharm.mssm.edu/Enrichr). The length of the bar represents the significance of that specific gene-set or term. The brighter the color, the more significant that term is. **(B)** Tissue specific expression analysis (TSEA) network for common genes showing enrichment for genes expressed in related tissues. **(C)** Tissue/cell enrichment of the PaGenBase category of Metascape. The darker the color, the more significant that term is. Colored bars correspond to tissues with an adjusted *p*-value ≤ 0.01.

We investigated the tissue and organ specific expression of the 274 unique genes. Several genes have enriched expressions in multiple organs, tissues, or cell types where they play a role in transcriptional regulation, epigenetic modification, differentiation and pathogenesis. Three online Bioinformatics tools, EnrichR web-tool (http://amp.pharm.mssm.edu/Enrichr), TSEA web-tool (http://genetics.wustl.edu/jdlab/tsea) and PaGenBase database in Metascape (http://metascape.org) were used to determine whether the identified genes are lung-specific or also expressed by other tissues. Through ARCHS4 resource in ErichR, cells of origin of identified genes were identified. Our analysis found that 150 of the identified genes (54.75%) are enriched in lung, spleen, colon bulk tissue and omentum as well as neutrophil and macrophages (Figure 5A). In TSEA web-tool, we found an over-representation of the 274 DEGs in the lung, blood, and adipose tissue (Figure 5B). The Pattern Gene Database (PaGenBase), which combines gene patterns from literature and data mining, was employed to identify tissue/cell-specific genes. We observe the same enriched pattern of expression in blood, lung, spleen and the heart (Figure 5C). This may suggest that dysregulated genes detected in the injured lung might recapitulate expression changes observed in spleen, heart, or colon of COVID-19 patients with pre-existing comorbidities.

### Functional enrichment analysis of unique and shared differentially expressed genes

**FIGURE 6:**
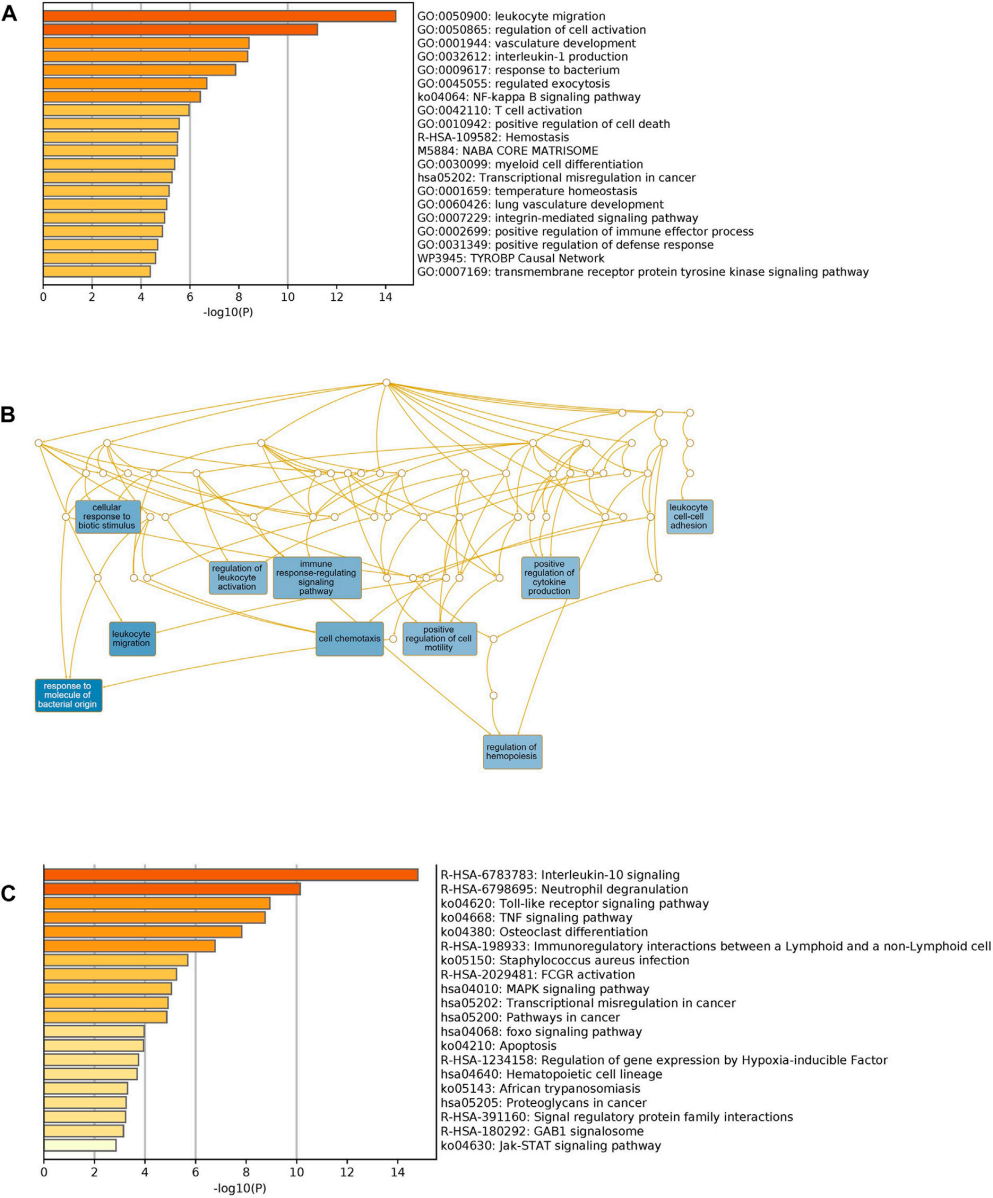
Functional enrichment of shared comorbidities-COVID-19 DEGs. **(A)** Bar graphs showing most significant biological processes. **(B)** Network illustrates the major non-redundant biological process. DEGs are enriched in biological processes related to leukocyte migration, T cell activation, regulation of hemopoiesis and integrin pathway. **(C)** Top Shared pathways; IL-10, TLRs and TNF signaling. The bar graphs were generated using Metascape (http://metascape.org) to illustrate the most enriched pathways and the Network was generated using WebGestalt (http://www.webgestalt.org/).

We used Metascape to generate a graphical representation of the specific enrichment of biological processes and pathways shared among COVID-19 infected patients with comorbidities. First, we interrogated the biological processes separately using unique to comorbidities (Supplementary Figure S1A) and to COVID-19 DEGs (Supplementary Figure S1B) to capture the pathways for each category. Secondly, we looked exclusively for pathways enrichment in KEGG and Reactome for unique comorbidities and COVID-19 gene sets, respectively (Supplementary Figures S2A,B). Shared biological processes and pathways were identified as well (Figures 6A,C). Major non-redundant biological process networks were also generated using WebGestalt (http://www.webgestalt.org/) (Figure 6B).

Unique genes for comorbidities alone were significantly enriched in the adaptive immune system, phagosome, neutrophil degranulation and signaling by interleukins. Unique genes for COVID-19 were also enriched in the adaptive immune system: signaling by Rho GTPases, heme biosynthesis and nervous system development. We observed, as expected, that shared pathways of identified genes were related to immune response, interleukin signaling and FCGR activation. Interestingly, among the top significantly enriched pathways, many are related to immunity processes such as response to bacteria, cytokines regulation and secretion of chemokines and regulation of leukocyte migration. Collectively these analyses support the role of the identified candidate genes in increasing the severity of clinical symptoms of COVID-19 infection among patients with pre-existing comorbidities.

### Molecular drug targets identification and similarity analysis with *ACE2* and *TMPRSS2*

**FIGURE 7:**
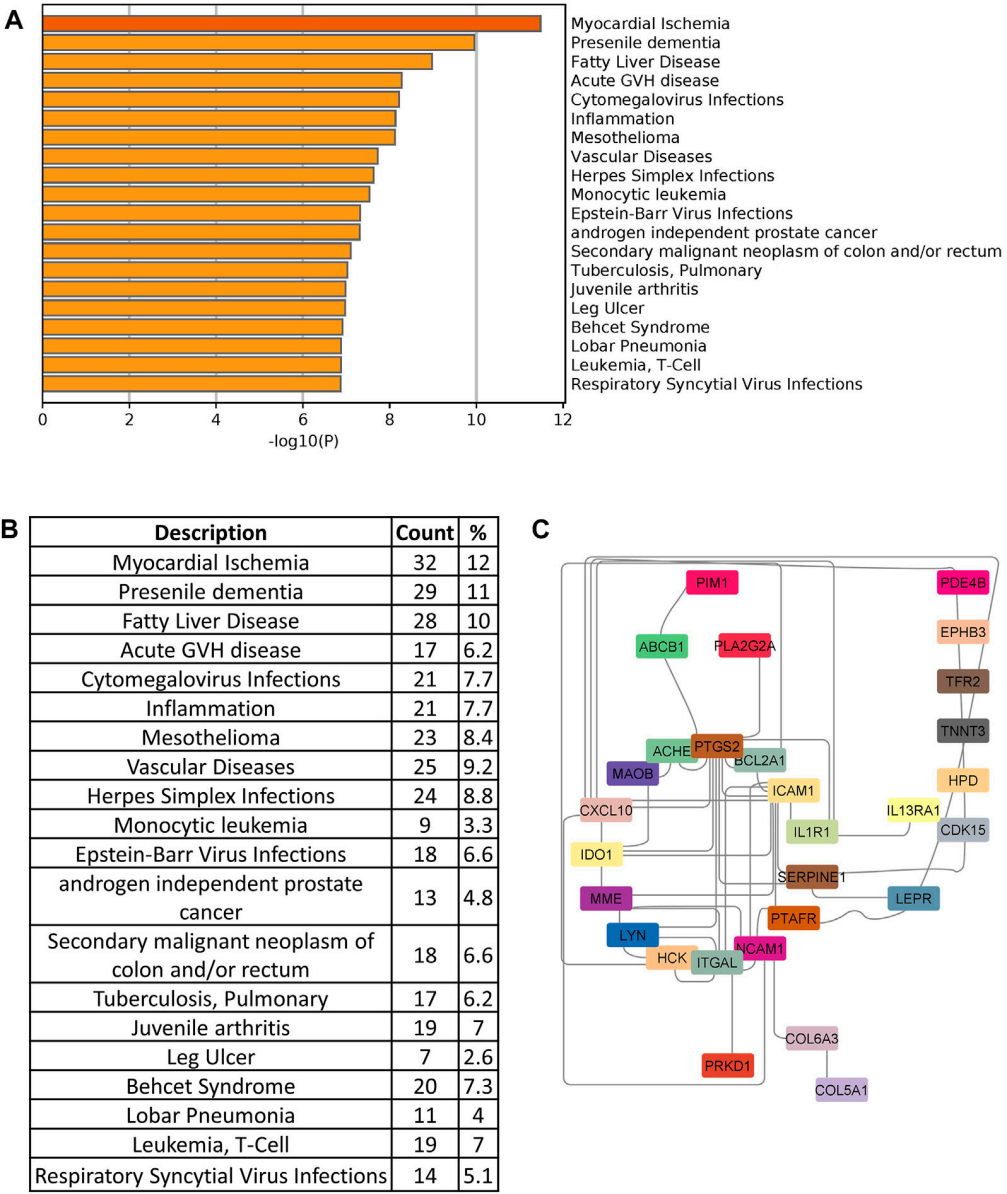
**(A)** Disease enrichment of shared genes of comorbidities and COVID-19 infection. Bar chart shows significant disease enrichment. **(B)** List of diseases related to comorbidities and COVID-19 with the percentage of specific common genes. **(C)** Network showing the specific drug targets.

In order to identify drug target, we searched the DisGeNet database to identify significant disease genes associated with comorbidities in Metascape. Myocardial ischemia, fatty liver disease, inflammation, vascular disease, and pneumonia were among the top disease classes represented in DisGeNet for all shared genes (Figure 7A). We identified 150 genes associated with top comorbidities (Figure 7B). This subset of genes was first submitted to the Open Targets Platform for druggability analysis and identification of potential novel drug targets, for existing drug repurposing against COVID-19 infection. Using the Open Targets Platform, 29 out of 150 genes were found to have known inhibitors/antagonists, modulators or agonists (Supplementary Table S2) and we prioritized these genes based on their association to top comorbidities (Figure 7C).

**FIGURE 8:**
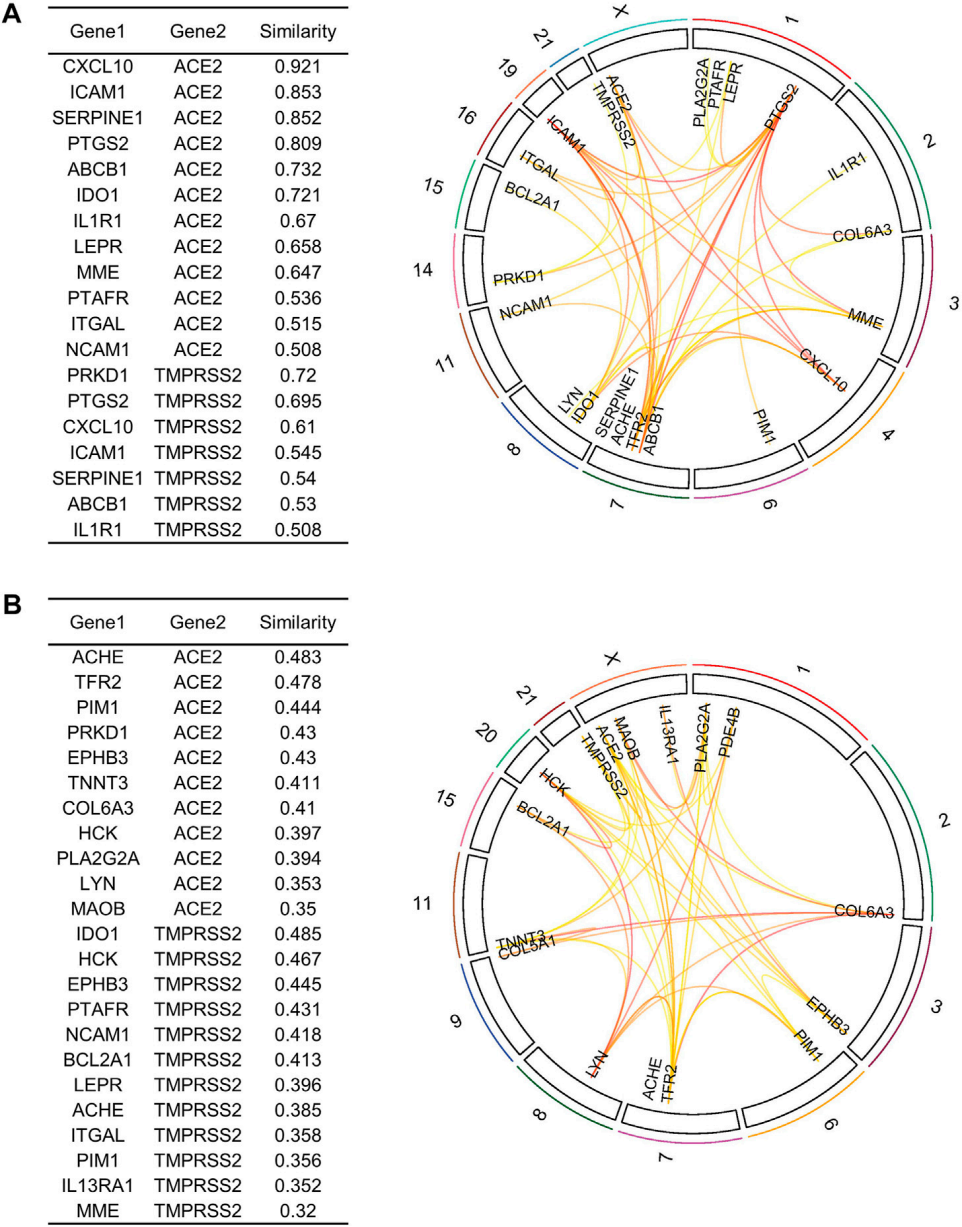
Similarity analysis and degree of relatedness of drug target genes to ACE2 and TMPRSS2 as a group to each other using eXploring Genomic Relations for enhanced interpretation. **(A)** ACE2 and TMPRSS2 showed a high degree of similarity with 13 out of the 29 identified genes. **(B)** Similarity analysis of the remaining 16 drug target genes. Drug targets were filtered based on cutoff score ≥ 0.5 as highly similar and ≤ 0.5 as slightly similar to ACE2 and TMPRSS2.

To further refine our drug target list, we performed a functional similarity analysis of the identified 29 target genes against Angiotensin-converting enzyme 2 (*ACE2*) and Transmembrane protease serine 2 (*TMPRSS2*), which play an important role in COVID-19 infection using the XGR web-tool for enhanced interpretation (http://galahad.well.ox.ac.uk:3020/). We found that 13 genes have a high similarity score (≥0.5) with *ACE2* and *TMPRSS2* (Figure 8A). Remaining 16 genes with a similarity score (≤0.5) were also shown (Figure 8B). Our analysis showed that some genes have similarities to both *ACE2* and *TMPRSS2*, but some are present only with *ACE2*, especially, *IDO1*, *MME*, *LEPR*, *ABCB1*, and *PTARF*. We found that only the *PRKD1* gene is similar to *TMPRSS2* and not to *ACE2* (Figure 8A). These results suggest that genes with the highest similarity to *ACE2* and *TMPRSS2* could influence the COVID-19 virus infection directly, leading to the possibility of molecular interaction and functional changes in severe COVID-19 patients. Furthermore, it may result in overexpression of *ACE2* and *TMPRSS2*, which explains the higher infection rate and disease severity in individuals with comorbidities. Drug target with high similarity scores to ACE2 and TMPRSS2 includes CXCL10, ICAM1, SERPINE1, PTGS2, ABCB1, IDO1, IL1R1, LEPR, MME, PTAFR, ITGAL, NCAM1 and PRKD1, (all up-regulated except *LEPR*) (Table 1) of which six targets overlap with PPI network top genes including ICAM1, PTGS2, CXCL10, ITGAL, NCAM1, SERPINE1 (Figure 4). Based on the association to top comorbidities and ACE2 and TMPRSS2 similarity scores, 17 drug target genes were identified, Marketed drugs for identified genes are indicated for the treatment of hypertension, heart failure, kidney failure, lung cancer, diabetes mellitus, emphysema, pulmonary obstruction, acute coronary syndrome, allergic rhinitis, inflammation, diabetic foot fever, diabetes mellitus, rheumatic disease and generalized lipodystrophy (Table 1). Identified drugs could be tested further for repurposing to reduce the severity risk among COVID-19-infected patients. Drug repurposing of marketed or approved drugs is an effective alternative method to uncover new indications rapidly.

**TABLE 1:** Suggested repurposable drugs retrieved from Open Targets platform.

Target	Drug	Mechanisms of action	Indications	Phase	Molecule type	Therapeutic area
Identified targets based on COVID-19 comorbidities-association as well as ACE2 and TMPRSS2 similarity score						
CXCL10^a^	ELDELUMAB	Inhibitor	Ulcerative colitis, Crohn’s disease, Rheumatoid arthritis	II	Antibody	Immune system disease
	NI-0801		Primary biliary cirrhosis	II		
			Allergic contact dermatitis	I		
IDO1	EPACADOSTAT	Inhibitor	Lung cancer	III	Small molecule	Lung cancer
	LINRODOSTAT					Respiratory or thoracic disease
						Cancer or benign tumor
LEPR	METRELEPTIN	Agonist	Lipodystrophy	IV	Small molecule	Immune system disease
			Liver disease			Nutritional or metabolic disease
			Obesity			
	CYCLOSPORINE	Modulator	Inflammation	IV		Immune system disease
			COVID-19	II		Infectious disease
	PREDNISOLONE	Agonist	Thrombocytopenia	IV	Small molecule	Respiratory or thoracic disease
			Asthma			Cardiovascular disease
			Myocarditis			
			COVID-19	III		
MME	SACUBITRIL	Inhibitor	Congestive heart failure	IV	Small molecule	Cardiovascular disease
			Myocardial infarction			Respiratory or thoracic disease
			COVID-19	I		
	ILEPATRIL		Hypertension	II		Cardiovascular disease
PTAFR	RUPATADINE	Antagonist	Allergy	III	Small molecule	Immune system disease
			Rheumatoid arthritis			
PTGS2^a^ ^,^ ^b^	ACEMETACIN	Inhibitor	Rheumatic disease	IV	Small molecule	Musculoskeletal or connective tissue disease
Targets based on COVID-19 comorbidities by association only						
MAOB^c^	SELEGILINE HYDROCHLORIDE	Inhibitor	Alzheimer’s disease	I	Small molecule	Psychiatric disorder
						Nervous system disease
	PARGYLINE		Hypertension	IV		Cardiovascular disease
	RASAGILINE MESYLATE		Parkinson’s disease	IV		Nervous system disease
PDE4B^d^	AMLEXANOX	Inhibitor	Airway obstruction	IV	Small molecule	Respiratory or thoracic disease
	DYPHYLLINE					
	DIPYRIDAMOLE		Stroke	IV		Cardiovascular disease
			Coronary artery disease			Infectious disease
			COVID-19	III		
	APREMILAST		Psoriasis	IV		Immune system disease
			COVID-19	III		Infectious disease
PLA2G2A	VARESPLADIB	Inhibitor	Coronary artery disease	II	Small molecule	Respiratory or thoracic disease
			Sickle cell anemia			Cardiovascular disease
			COVID-19			Hematologic disease
	VARESPLADIB METHYL		Acute coronary syndrome	III		Respiratory or thoracic disease
			Kidney disease	I		Cardiovascular disease
COL5A1	COLLAGENASE CLOSTRIDIUM HISTOLYTICUM	Hydrolytic Enzyme	Skin ulcer	IV	Enzyme	Integumentary system disease
						Cardiovascular disease
	OCRIPLASMIN	Hydrolytic Enzyme	Eye disease	IV		Nervous system disease
		Proteolytic Enzyme	Stroke	II		Cardiovascular disease
Targets based on ACE2 and TMPRSS2 similarity scores only						
IL1R1	ANAKINRA	Antagonist	Rheumatoid arthritis	IV	Small molecule	Immune system disease
			Pericarditis	III		
			COVID-19			
ABCB1	ZOSUQUIDAR	Inhibitor	Leukemia	III	Small molecule	Immune system disease
	TARIQUIDAR	Inhibitor	Lung cancer	III	Small molecule	respiratory or thoracic disease, cancer or benign tumor
ICAM1^a^	ALICAFORSEN	Antisense inhibitor	Crohn’s disease	III	Small molecule	Immune system disease, Gastrointestinal disease
ITGAL^a^	EFALIZUMAB	Inhibitor	Immune system disease	IV	Antibody	immune system disease
	LIFITEGRAST	Antagonist	Dry eye syndrome	IV	Small molecule	Immune system disease, Disorder of visual system
PRKD1	MIDOSTAURIN	Inhibitor	Systemic mastocytosis	IV	Small molecule	Immune system disease, Hematologic disease
SERPINE1^a^	ALEPLASININ	Inhibitor	Alzheimer disease	I	Small molecule	Nervous system disease
NCAM1^a^	LORVOTUZUMAB MERTANSINE	Inhibitor	leukemia	II	Antibody	Immune system disease, Hematologic disease

^a^ Drug target that overlapped with PPI network top genes.

^b^ A total of 69 drug phase III and IV were identified from Open Targets platform as inhibitors of PTGS2. Eight drugs, including ASPIRIN, IBUPROFEN, INDOMETHACIN, ACETAMINOPHEN, NAPROXEN, CELECOXIB, DICLOFENAC SODIUM and DICLOFENAC, are indicated in COVID-19.

^c^ A total of 13 drug phase III and IV were identified from Open Targets platform as inhibitors of MAOB.

^d^ A total of 18 drug phase III and IV were identified from Open Targets platform as inhibitors of PDE4B.

### Signaling network analysis

The signaling causal interaction network (in SIGNOR 3.0) of the candidate genes showed that 15 out of 17 genes were central regulators of multiple signaling pathways (Supplementary Figure S3). Dysregulated pathways includes inflammation, immune response, chemotaxis, fibrinolysis, cell adhesion and acute respiratory distress syndrome (ARDS). Most genes like *PTGS2*, *PTAFR*, MOAB, PRKD1, ABCB1, IL1R1 and *LEPR* showed high interaction (with >8 connectivity). Of note, *IDO1* and *PLA2G2A* were not present in the SIGNOR 3.0 database.

## Phenotypic implications of the identified genes

Finally, we investigated the overall phenotypic association of shared genes. We performed our analysis using ToppGene (https://toppgene.cchmc.org/) for mouse phenotype analysis. We found a consistent phenotype pattern with the gene ontology and pathway enrichment, including abnormal innate and adaptive immunity, abnormal macrophage production, pulmonary interstitial fibrosis, and decreased inflammatory response (Supplementary Figure S4A). We also performed the phenotypic association of the 17 candidate genes from Open Target Platform analysis, to increase the specificity of observed phenotypes. In addition to the above-mentioned phenotypes, candidate genes were highly associated with abnormal type IV hypersensitivity reaction, abnormal body temperature, abnormal interleukin level and altered susceptibility to infection (Supplementary Figure S4B).

## Discussion

The discovery of new biomarkers that can differentiate and categorize distinct COVID-19 phenotypes is urgently needed. Elucidating specific biomarkers to severe COVID-19 disease phenotype allows early intervention that may improve the outcome. Patients with pre-existing comorbidities, including cardiovascular disease, obesity, diabetes, and respiratory system diseases, are at a greater risk of developing severe symptoms or fatal with COVID-19 infection. Chronic diseases share risk factors or disease pathways with infectious disorders, like the pro-inflammatory state and the attenuation of the innate immune response (Yang et al., 2020a).

Differential gene expression analysis between patients and healthy control groups is the common logical method utilized to identify disease-associated changes. Here, we used transcriptomic data integration, differential expression analysis, and co-expression network analysis to identify underlying genetic factors and elucidate potential target drug or biomarkers that might explain the contribution of the comorbidities to increase the severity of COVID-19 infection symptoms by molecular interactions. Data for other respiratory illnesses such as influenza virus, RSV and MERS were intersected with COVID-19 infection DEGs to remove the common viral pathway genes, which otherwise will be tagged as shared biomarkers for comorbidities-associated severe COVID-19 symptoms.

### Comorbidities-COVID-19 pathogenicity

We identified 274 significant DEGs (|log2FC| >1.5; *p* < 0.05) shared between comorbidities and COVID-19. Functional analysis of shared genes among both comorbidities and COVID-19 infection revealed a critical involvement and significant enrichment in immune response processes including leukocyte migration, interleukin production, T cell activation, lung vasculature development, cell death and temperature homeostasis (Figure 5). Shared pathways are mostly related to interleukin-10, TNF and FOXO signaling which are involved in cellular processes like apoptosis, cell cycle, control of cell cycle glucose metabolism and oxidative stress resistance (Figure 5C).

Based on PPI, biological process and molecular pathway enrichments, we found that both comorbidities and COVID-19 have a strong association with symptomatically related diseases such as early-onset dementia, fatty liver disease, mesothelioma, vascular diseases, cytomegalovirus, herpes simplex and Epstein–Barr Virus infections (Figure 6). COVID-19 infection causes a severe multisystem inflammatory disease in critically ill patients consisting of respiratory distress, cardiovascular dysfunction, thrombosis, neurological manifestations, dysregulated inflammatory process and fibrosis. The strong link of shared genes with several diseases can be attributed to the critical role of the identified genes.

### *ACE2* and *TMPRSS2* correlation and target genes drug repurposing

*ACE2* and *TMPRSS2* are the two major host receptors which contribute to the virulence and the severe pathogenesis of COVID-19. Our computational analysis of the PPI network identified several highly interacting genes such as ICAM1, PTGS2, CXCL10, ITGAL, NCAM1, SERPINE1, IDO1, LEPR, PTAFR, and PLA2G2A (Figure 4)*,* which also showed a higher degree of functional similarity with *ACE2* and *TMPRSS2* (Figure 7) suggesting a potential direct or indirect interaction with the COVID-19 spike protein (Cuperlovic-Culf et al., 2021).

Systemic inflammation is a hallmark of many infectious diseases including COVID-19. An appropriate inflammatory response is essential for eradicating pathogens. Prolonged or excessive inflammatory response is clearly detrimental and fatal to the host if not carefully managed. Identified biomarkers could be categorized as acute phase reacting immunomodulators as they significantly alter the inflammatory cells’ responses, which trigger the pro-inflammatory signaling pathway (Hata and Breyer, 2004; Vazirinejad et al., 2014; Guo et al., 2019; Kiernan and MacIver, 2020).

Identified biomarkers were found to be involved in innate immunity, mostly in monocyte and macrophage function and are essential for immune cell homeostasis. Their early activation/inactivation results in uncontrolled production, perturbation, and inadequate recruitment of active immune cells, which in turn promotes the cellular stress, resident cell’s damage and, eventually, organ failure. The interplay of these main biomarkers during infection induced early immune response are discussed elsewhere (Fang et al., 2020; Goyal et al., 2020; Grasselli et al., 2020; Holman et al., 2020; Madjid et al., 2020; Shi et al., 2020; Wu and McGoogan, 2020; Zhou et al., 2020).

Dysregulation of these COVID-19 acute phase pro-inflammatory genes can greatly induce a detrimental effect by prolonging the inflammatory response, delayed viral clearance and thus increasing the risk of severe and prolonged COVID-19 symptoms (Coperchini et al., 2021) For example, only augmented *CXCL10* plasma levels in COVID-19 infected patients were found to be significantly correlated with the viral load and disease severity (Guo et al., 2022a; Guo et al., 2022b) and higher mortality, especially in males (Draxler et al., 2017). It is worth noting that *CXCL10* is not expressed in healthy individuals and was significantly lower in asymptomatic cases than in symptomatic patients. Furthermore, COVID-19 patients have delayed or slow immune dysfunction response, making these patients more susceptible to suffer from exacerbation and impaired lung function (Danladi and Sabir, 2021). Increased expression of *PDE4B* results in a pro-inflammatory phenotype in neutrophils and macrophages (Ulm et al., 2013). Selective inhibitors in the knockout mouse model for severe chronic obstructive pulmonary disorder (COPD) have established the beneficial anti-inflammatory effect of *PDE4B* to improve lung function and decrease exacerbation rates. Specific inhibition of these factors significantly alters the functions of specialized inflammatory cells suggesting that these factors may help in early diagnosis and could be predictors of clinical symptoms (Guo et al., 2022a). *IDO1* is also an important immunomodulatory gene in T lymphocyte activation and in modulating immune tolerance (Veazey et al., 2020; Makaremi et al., 2022). In preclinical models, pharmacological inhibitors of *IDO1* have therapeutic utility in various diseases, including cancer, HIV and influenza (Yang et al., 2020b; Chang et al., 2020; Chi et al., 2020). Interestingly, *IDO1* inhibitors suppress the COVID-19-induced pro-inflammatory cytokine release, including TNF-α, IL-6, IL-1α and IL-1β in isolated PBMCs derived from COVID-19-infected rhesus macaques and lower mortality among critically ill patients with COVID-19 (Song et al., 2020; Agrawal et al., 2021). This confirms the critical role of the *IDO1* gene network in COVID-19-exaggerated cytokine release. Moreover, *MAOB*, *ICAM1, SERPINE1, MME, IL1R1, PLA2G2A, LEPR* and *PTGS2* have emerged as attractive pharmaceutical targets for COVID-19 infection treatment recently (Puccetti and Grohmann, 2007; Iyer et al., 2018; Blair et al., 2019; Tibbo and Baillie, 2020).

Our analysis also showed that *LEPR* is downregulated in COVID-19. The expression of *LEPR* is inversely related to leptin concentrations (Potula et al., 2005). *LEPR* has been shown to influence both innate and adaptive immune responses (Singh and Salunke, 2021). Several studies on mouse models of viral infection addressed the role of *LEPR* and leptin in the pathogenesis of infectious diseases (Potula et al., 2005). Interestingly, global reduction of the *LEPR*s in obese mice demonstrated impaired influenza virus viral clearance and reduced survival (Group et al., 2021). Additionally, *LEPR* deficient mouse exhibits increased susceptibility to respiratory infections suggesting a leptin requirement in the pulmonary innate and adaptive immune response to infection (Tomazini et al., 2020).

Arterial thrombosis in the lung and other organs has been reported in critically ill COVID-19 patients (Bellis et al., 2020). Platelet Activating Factor Receptor (*PTAFR* or PAFR) is exploited by respiratory viruses and bacteria to interact with human cells and initiate infection (Cauchois et al., 2020). It was implicated in the entry of H1N1 and H3N2, Dengue and respiratory syncytial viruses (Mohammed El Tabaa and Mohammed El Tabaa, 2020). *PTAFR* deficient mice, as well as wild-type mice treated with a *PTAFR* antagonist, had less pulmonary inflammation and injury and reduced lethality rates when infected with Influenza A virus (Mohammed El Tabaa and Mohammed El Tabaa, 2020). These findings show that *PTAFR* is a major driver of exacerbated inflammation during viral infection. Inhibition of *PTAFR* offers significant protection against fatal outcomes and lung injury associated with the flu. *PLA2G2A* is a potent mediator of the inflammatory process and also proatherogenic, implicated in various clinical conditions, including sepsis (Ostadkarampour and Putnins, 2021). Increased *PLA2G2A* levels is a significant risk factor for coronary artery disease and are found to be highly associated with disease severity in COVID-19 patients (Lahlou et al., 2002). It will be interesting to explore the potential of these genes as early systemic diagnostic biomarkers for COVID-19.

Although our study aims to map key genes and pathway alterations in patients with COVID-19 and comorbidities, our results at present are observational and there are limitations to our study. First, there was a lack of data from COVID-19 patients with comorbidities and present COVID-19 datasets were mostly published on 2020 before the emergence of Omicron variant. Second, there was a lack of data on COVID-19 variants effect in the present study, which may become a focus of future studies as more datasets are put in the public domain. That will allow the scientists to explore the effect of different COVID-19 variants into the specific molecular mechanism and to the clinical severity of COVID-19. Despite these disadvantages, the present study was powered by integrated bioinformatics analysis identified DEGs that exhibited large-fold changes with high statistical significance. Moreover, there was consistency between our findings and the previous studies. Hence, the results of our analysis could be reliable to a large extent and explain the specific role of these genes in COVID-19 and indirectly give insights into how such genes contribute to the severity of COVID-19 infection. Lastly, there was a lack of experimental validation for these findings in the present study, which may become a focus of future studies.

## Conclusion

In summary, we used publicly available gene expression profiles from severe/deceased COVID-19 infected individuals and patients with comorbidities alone — CVD, atherosclerosis, diabetes, and obesity, to identify overlapping key genes associated with the severity of COVID-19 infection. We identified 29 genes shared between severe COVID-19 and comorbidities. Our results show that some of the available drugs against the identified genes are already in use as COVID-19 therapy, suggesting that identified genes as clinical indicators for COVID-19 disease severity, central drivers of immune deficit and multiorgan failure. We recommend that existing drugs for identified genes can be investigated further for their therapeutic efficacy and repurposed to treat COVID-19 patients with severe symptoms.

## Data Availability

The datasets presented in this study can be found in online repositories. The names of the repository/repositories and accession number(s) can be found in the article/Supplementary Material.

## References

[B1] Agrawal S. Salazar J. Tran T. M. Agrawal A. (2021). Sex-related differences in innate and adaptive immune responses to SARS-CoV-2. Front. Immunol. 12, 739757. 10.3389/fimmu.2021.739757 34745109 PMC8563790

[B2] Alti D. Sambamurthy C. Kalangi S. K. (2018). Emergence of leptin in infection and immunity: Scope and challenges in vaccines formulation. Front. Cell. Infect. Microbiol. 8, 147. 10.3389/fcimb.2018.00147 29868503 PMC5954041

[B3] Banaganapalli B. Al-Rayes N. Awan Z. A. Alsulaimany F. A. Alamri A. S. Elango R. (2021). Multilevel systems biology analysis of lung transcriptomics data identifies key miRNAs and potential miRNA target genes for SARS-CoV-2 infection. Comput. Biol. Med. 135, 104570. 10.1016/j.compbiomed.2021.104570 34157472 PMC8197616

[B4] Banaganapalli B. Mansour H. Mohammed A. Alharthi A. M. Aljuaid N. M. Nasser K. K. (2020). Exploring celiac disease candidate pathways by global gene expression profiling and gene network cluster analysis. Sci. Rep. 10, 16290. 10.1038/s41598-020-73288-6 33004927 PMC7529771

[B5] Bellis A. Mauro C. Barbato E. Trimarco B. Morisco C. (2020). The rationale for Angiotensin receptor neprilysin inhibitors in a multi-targeted therapeutic approach to COVID-19. Int. J. Mol. Sci. 21, E8612. 10.3390/ijms21228612 PMC7696732 33203141

[B6] Blair A. B. Kleponis J. Thomas D. L. 2nd Muth S. T. Murphy A. G. Kim V. (2019). Ido1 inhibition potentiates vaccine-induced immunity against pancreatic adenocarcinoma. J. Clin. Invest. 129, 1742–1755. 10.1172/JCI124077 30747725 PMC6436883

[B7] Cauchois R. Koubi M. Delarbre D. Manet C. Carvelli J. Blasco V. B. (2020). Early IL-1 receptor blockade in severe inflammatory respiratory failure complicating COVID-19. Proc. Natl. Acad. Sci. U. S. A. 117, 18951–18953. 10.1073/pnas.2009017117 32699149 PMC7430998

[B8] Chang Mo G. Yuan X. Tao Y. Peng X. Wang F. S. Xie L. (2020). Time kinetics of viral clearance and resolution of symptoms in novel coronavirus infection. Am. J. Respir. Crit. Care Med. 201, 1150–1152. 10.1164/rccm.202003-0524LE 32200654 PMC7193851

[B9] Chi Y. Ge Y. Wu B. Zhang W. Wu T. Wen T. (2020). Serum cytokine and chemokine profile in relation to the severity of coronavirus disease 2019 in China. J. Infect. Dis. 222, 746–754. 10.1093/infdis/jiaa363 32563194 PMC7337752

[B10] Coperchini F. Chiovato L. Rotondi M. (2021). Interleukin-6, CXCL10 and infiltrating macrophages in COVID-19-related cytokine storm: Not one for all but all for one. Front. Immunol. 12, 668507. 10.3389/fimmu.2021.668507 33981314 PMC8107352

[B11] Cuperlovic-Culf M. Cunningham E. L. Teimoorinia H. Surendra A. Pan X. Bennett S. A. L. (2021). Cerebrospinal fluid spermidine, glutamine and putrescine predict postoperative delirium following elective orthopaedic surgery. Sci. Rep. 11, 4191. 10.1038/s41598-019-40544-3 30862889 PMC6414730

[B12] Danladi J. Sabir H. (2021). Innate immunity, inflammation activation and heat-shock protein in COVID-19 pathogenesis. J. Neuroimmunol. 358, 577632. 10.1016/j.jneuroim.2021.577632 34186336 PMC8196476

[B13] Dolan M. E. Hill D. P. Mukherjee G. McAndrews M. S. Chesler E. J. Blake J. A. (2020). Investigation of COVID-19 comorbidities reveals genes and pathways coincident with the SARS-CoV-2 viral disease. Sci. Rep. 10, 20848. 10.1038/s41598-020-77632-8 33257774 PMC7704638

[B14] Dore E. Boilard E. (2019). Roles of secreted phospholipase A2 group IIA in inflammation and host defense. Biochim. Biophys. Acta. Mol. Cell Biol. Lipids 1864, 789–802. 10.1016/j.bbalip.2018.08.017 30905346

[B15] Draxler D. F. Sashindranath M. Medcalf R. L. (2017). Plasmin: A modulator of immune function. Semin. Thromb. Hemost. 43, 143–153. 10.1055/s-0036-1586227 27677178

[B16] Facchiano A. Facchiano F. Facchiano A. (2020). An investigation into the molecular basis of cancer comorbidities in coronavirus infection. FEBS Open Bio 10, 2363–2374. 10.1002/2211-5463.12984 PMC7537529 32970391

[B17] Fang L. Karakiulakis G. Roth M. (2020). Are patients with hypertension and diabetes mellitus at increased risk for COVID-19 infection? Lancet. Respir. Med. 8, e21. 10.1016/S2213-2600(20)30116-8 32171062 PMC7118626

[B18] Fox T. Ruddiman K. Lo K. B. Peterson E. DeJoy R. 3rd Salacup G. (2021). The relationship between diabetes and clinical outcomes in COVID-19: A single-center retrospective analysis. Acta Diabetol. 58, 33–38. 10.1007/s00592-020-01592-8 32804317 PMC7429932

[B19] Garcia C. C. Russo R. C. Guabiraba R. Fagundes C. T. Polidoro R. B. Tavares L. P. (2010). Platelet-activating factor receptor plays a role in lung injury and death caused by Influenza A in mice. PLoS Pathog. 6, e1001171. 10.1371/journal.ppat.1001171 21079759 PMC2974216

[B20] Goyal P. Choi J. J. Pinheiro L. C. Schenck E. J. Chen R. Jabri A. (2020). Clinical characteristics of covid-19 in New York city. N. Engl. J. Med. 382, 2372–2374. 10.1056/NEJMc2010419 32302078 PMC7182018

[B21] Grasselli G. Zangrillo A. Zanella A. Antonelli M. Cabrini L. Castelli A. (2020). Baseline characteristics and outcomes of 1591 patients infected with SARS-CoV-2 admitted to ICUs of the lombardy region, Italy. JAMA 323, 1574–1581. 10.1001/jama.2020.5394 32250385 PMC7136855

[B22] Group R. C. Horby P. Lim W. S. Emberson J. R. Mafham M. Bell J. L. (2021). Dexamethasone in hospitalized patients with covid-19. N. Engl. J. Med. Overseas. Ed. 384, 693–704. 10.1056/nejmoa2021436 PMC7383595 32678530

[B23] Guo G. Sun L. Yang L. Xu H. (2019). Ido1 depletion induces an anti-inflammatory response in macrophages in mice with chronic viral myocarditis. Cell Cycle 18, 2598–2613. 10.1080/15384101.2019.1652471 31416389 PMC6773230

[B24] Guo L. Schurink B. Roos E. Nossent E. J. Duitman J. W. Vlaar A. P. (2022). Indoleamine 2, 3-dioxygenase (Ido)-1 and Ido-2 activity and severe course of COVID-19. J. Pathol. 256, 256–261. 10.1002/path.5842 34859884 PMC8897979

[B25] Guo Z. Yang H. Zhang J-R. Zeng W. Hu X. (2022). Leptin receptor signaling sustains metabolic fitness of alveolar macrophages to attenuate pulmonary inflammation. Sci. Adv. 8, eabo3064. 10.1126/sciadv.abo3064 35857512 PMC9286500

[B26] Hata A. N. Breyer R. M. (2004). Pharmacology and signaling of prostaglandin receptors: Multiple roles in inflammation and immune modulation. Pharmacol. Ther. 103, 147–166. 10.1016/j.pharmthera.2004.06.003 15369681

[B27] Hernandez-Garduno E. (2020). Obesity is the comorbidity more strongly associated for Covid-19 in Mexico. A case-control study. Obes. Res. Clin. Pract. 14, 375–379. 10.1016/j.orcp.2020.06.001 32536475 PMC7290168

[B28] Holman N. Knighton P. Kar P. O'Keefe J. Curley M. Weaver A. (2020). Risk factors for COVID-19-related mortality in people with type 1 and type 2 diabetes in england: A population-based cohort study. Lancet. Diabetes Endocrinol. 8, 823–833. 10.1016/S2213-8587(20)30271-0 32798471 PMC7426091

[B29] Hottz E. D. Azevedo-Quintanilha I. G. Palhinha L. Teixeira L. Barreto E. A. Pao C. R. R. (2020). Platelet activation and platelet-monocyte aggregate formation trigger tissue factor expression in patients with severe COVID-19. Blood 136, 1330–1341. 10.1182/blood.2020007252 32678428 PMC7483437

[B30] Iyer S. S. Gensollen T. Gandhi A. Oh S. F. Neves J. F. Collin F. (2018). Dietary and microbial oxazoles induce intestinal inflammation by modulating aryl hydrocarbon receptor responses. Cell 173, 1123–1134. 10.1016/j.cell.2018.04.037 29775592 PMC6119676

[B31] Kiernan K. MacIver N. J. (2020). The role of the adipokine leptin in immune cell function in health and disease. Front. Immunol. 11, 622468. 10.3389/fimmu.2020.622468 33584724 PMC7878386

[B32] Lahlou N. Issad T. Lebouc Y. Carel J. C. Camoin L. Roger M. (2002). Mutations in the human leptin and leptin receptor genes as models of serum leptin receptor regulation. Diabetes 51, 1980–1985. 10.2337/diabetes.51.6.1980 12031989

[B33] Madjid M. Safavi-Naeini P. Solomon S. D. Vardeny O. (2020). Potential effects of coronaviruses on the cardiovascular system: A review. JAMA Cardiol. 5, 831–840. 10.1001/jamacardio.2020.1286 32219363

[B34] Makaremi S. Asgarzadeh A. Kianfar H. Mohammadnia A. Asghariazar V. Safarzadeh E. (2022). The role of IL-1 family of cytokines and receptors in pathogenesis of COVID-19. Inflamm. Res. 71, 923–947. 10.1007/s00011-022-01596-w 35751653 PMC9243884

[B35] Malik P. Patel U. Patel K. Martin M. Shah C. Mehta D. (2021). Obesity a predictor of outcomes of COVID-19 hospitalized patients-A systematic review and meta-analysis. J. Med. Virol. 93, 1188–1193. 10.1002/jmv.26555 32975814 PMC7537321

[B36] Martin-Romero C. Santos-Alvarez J. Goberna R. Sanchez-Margalet V. (2000). Human leptin enhances activation and proliferation of human circulating T lymphocytes. Cell. Immunol. 199, 15–24. 10.1006/cimm.1999.1594 10675271

[B37] Mohammed El Tabaa M. Mohammed El Tabaa M. (2020). Targeting neprilysin (nep) pathways: A potential new hope to defeat COVID-19 ghost. Biochem. Pharmacol. 178, 114057. 10.1016/j.bcp.2020.114057 32470547 PMC7250789

[B38] Mujalli A. Banaganapalli B. Alrayes N. M. Shaik N. A. Elango R. Al-Aama J. Y. (2020). Myocardial infarction biomarker discovery with integrated gene expression, pathways and biological networks analysis. Genomics 112, 5072–5085. 10.1016/j.ygeno.2020.09.004 32920122

[B39] Ostadkarampour M. Putnins E. E. (2021). Monoamine oxidase inhibitors: A review of their anti-inflammatory therapeutic potential and mechanisms of action. Front. Pharmacol. 12, 676239. 10.3389/fphar.2021.676239 33995107 PMC8120032

[B40] Potula R. Poluektova L. Knipe B. Chrastil J. Heilman D. Dou H. (2005). Inhibition of indoleamine 2, 3-dioxygenase (Ido) enhances elimination of virus-infected macrophages in an animal model of HIV-1 encephalitis. Blood 106, 2382–2390. 10.1182/blood-2005-04-1403 15961516 PMC1895260

[B41] Puccetti P. Grohmann U. (2007). Ido and regulatory T cells: A role for reverse signalling and non-canonical NF-kappaB activation. Nat. Rev. Immunol. 7, 817–823. 10.1038/nri2163 17767193

[B42] Radigan K. A. Morales-Nebreda L. Soberanes S. Nicholson T. Nigdelioglu R. Cho T. (2014). Impaired clearance of influenza A virus in obese, leptin receptor deficient mice is independent of leptin signaling in the lung epithelium and macrophages. PLoS One 9, e108138. 10.1371/journal.pone.0108138 25232724 PMC4169489

[B43] Rijneveld A. W. Weijer S. Florquin S. Speelman P. Shimizu T. Ishii S. (2004). Improved host defense against pneumococcal pneumonia in platelet-activating factor receptor-deficient mice. J. Infect. Dis. 189, 711–716. 10.1086/381392 14767826

[B44] Shi S. Qin M. Shen B. Cai Y. Liu T. Yang F. (2020). Association of cardiac injury with mortality in hospitalized patients with COVID-19 in wuhan, China. JAMA Cardiol. 5, 802–810. 10.1001/jamacardio.2020.0950 32211816 PMC7097841

[B45] Singh M. K. Mobeen A. Chandra A. Joshi S. Ramachandran S. (2021). A meta-analysis of comorbidities in COVID-19: Which diseases increase the susceptibility of SARS-CoV-2 infection? Comput. Biol. Med. 130, 104219. 10.1016/j.compbiomed.2021.104219 33486379 PMC7836641

[B46] Singh R. Salunke D. B. (2021). Diverse chemical space of indoleamine-2, 3-dioxygenase 1 (Ido1) inhibitors. Eur. J. Med. Chem. 211, 113071. 10.1016/j.ejmech.2020.113071 33341650

[B47] Snider J. M. You J. K. Wang X. Snider A. J. Hallmark B. Seeds M. C. (2021). Group iia secreted phospholipase A 2 plays a central role in the pathobiology of COVID-19. medRxiv. 10.1101/2021.02.22.21252237 PMC8483752 34428181

[B48] Song C. Y. Xu J. He J. Q. Lu Y. Q. (2020). Immune dysfunction following COVID-19, especially in severe patients. Sci. Rep. 10, 15838. 10.1038/s41598-020-72718-9 32985562 PMC7522270

[B49] Tibbo A. J. Baillie G. S. (2020). Phosphodiesterase 4B: Master regulator of brain signaling. Cells 9, E1254. 10.3390/cells9051254 PMC7291338 32438615

[B50] Tomazini B. M. Maia I. S. Cavalcanti A. B. Berwanger O. Rosa R. G. Veiga V. C. (2020). Effect of dexamethasone on days alive and ventilator-free in patients with moderate or severe acute respiratory distress syndrome and COVID-19: The CoDEX randomized clinical trial. JAMA 324, 1307–1316. 10.1001/jama.2020.17021 32876695 PMC7489411

[B51] Ulm C. Saffarzadeh M. Mahavadi P. Müller S. Prem G. Saboor F. (2013). Soluble polysialylated NCAM: A novel player of the innate immune system in the lung. Cell. Mol. Life Sci. 70, 3695–3708. 10.1007/s00018-013-1342-0 23619613 PMC11113884

[B52] Vazirinejad R. Ahmadi Z. Kazemi Arababadi M. Hassanshahi G. Kennedy D. (2014). The biological functions, structure and sources of CXCL10 and its outstanding part in the pathophysiology of multiple sclerosis. Neuroimmunomodulation 21, 322–330. 10.1159/000357780 24642726

[B53] Veazey J. M. Eliseeva S. I. Hillman S. E. Stiles K. Smyth T. R. Morrissey C. E. (2020). Inhibiting protein kinase D promotes airway epithelial barrier integrity in mouse models of influenza A virus infection. Front. Immunol. 11, 580401. 10.3389/fimmu.2020.580401 33381112 PMC7767883

[B54] Wu Z. McGoogan J. M. (2020). Characteristics of and important lessons from the coronavirus disease 2019 (COVID-19) outbreak in China: Summary of a report of 72314 cases from the Chinese center for disease control and prevention. JAMA 323, 1239–1242. 10.1001/jama.2020.2648 32091533

[B55] Yang J. Zheng Y. Gou X. Pu K. Chen Z. Guo Q. (2020). Prevalence of comorbidities and its effects in patients infected with SARS-CoV-2: A systematic review and meta-analysis. Int. J. Infect. Dis. 94, 91–95. 10.1016/j.ijid.2020.03.017 32173574 PMC7194638

[B56] Yang Y. Shen C. Li J. Yuan J. Wei J. Huang F. (2020). Plasma IP-10 and MCP-3 levels are highly associated with disease severity and predict the progression of COVID-19. J. Allergy Clin. Immunol. 146, 119–127. 10.1016/j.jaci.2020.04.027 32360286 PMC7189843

[B57] Zhou Y. Yang Q. Chi J. Dong B. Lv W. Shen L. (2020). Comorbidities and the risk of severe or fatal outcomes associated with coronavirus disease 2019: A systematic review and meta-analysis. Int. J. Infect. Dis. 99, 47–56. 10.1016/j.ijid.2020.07.029 32721533 PMC7381888

