# Supplementary figures and images for: Bioinformatics insights into the genes and pathways on severe COVID-19 pathology in patients with comorbidities
Source: Front Physiol. 2022 Dec 14;13:1045469. doi: 10.3389/fphys.2022.1045469 (PMC9795193; doi:10.3389/fphys.2022.1045469)

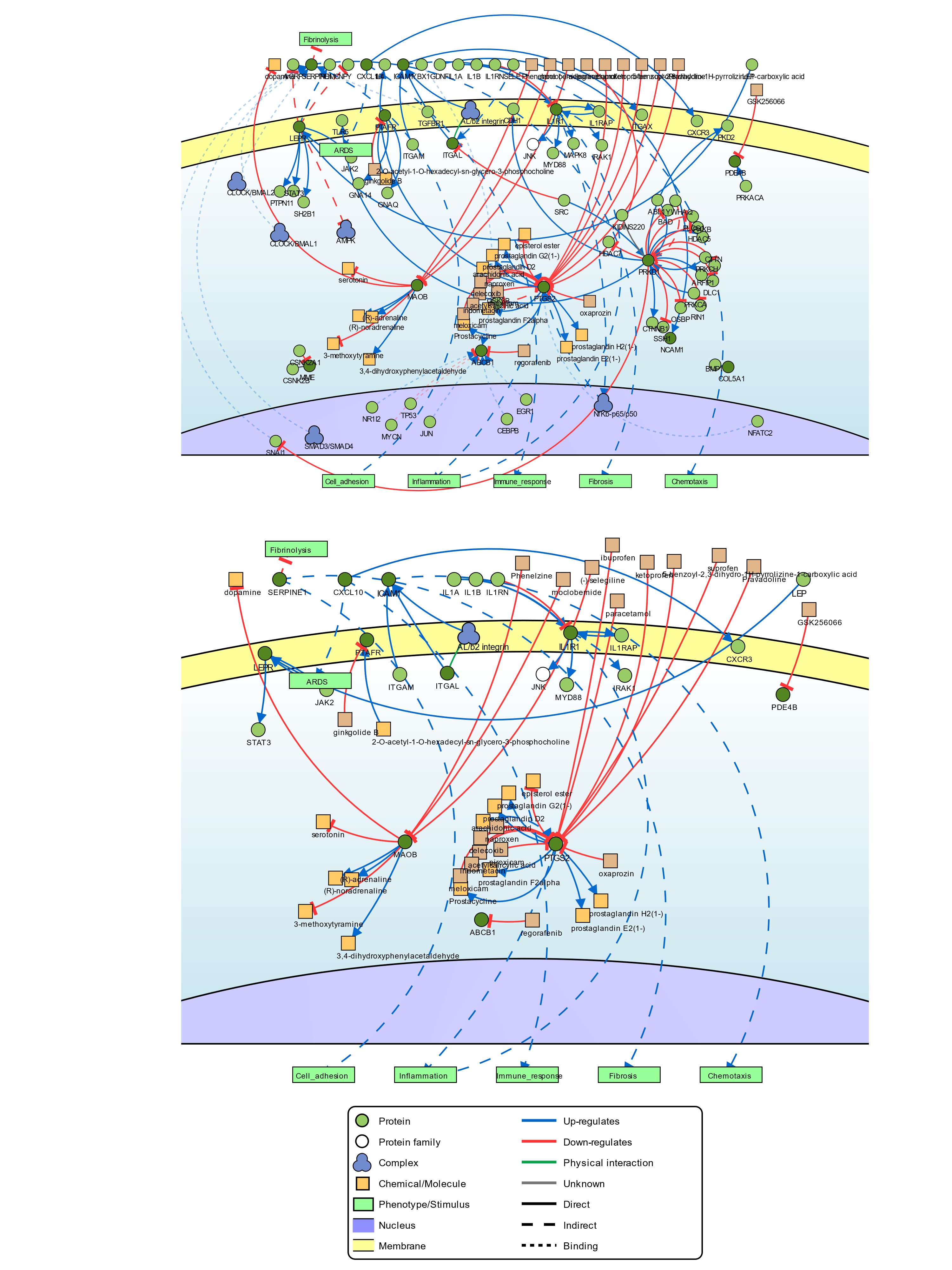

Supplement: Supplementary file 2 [file Image3.JPEG]

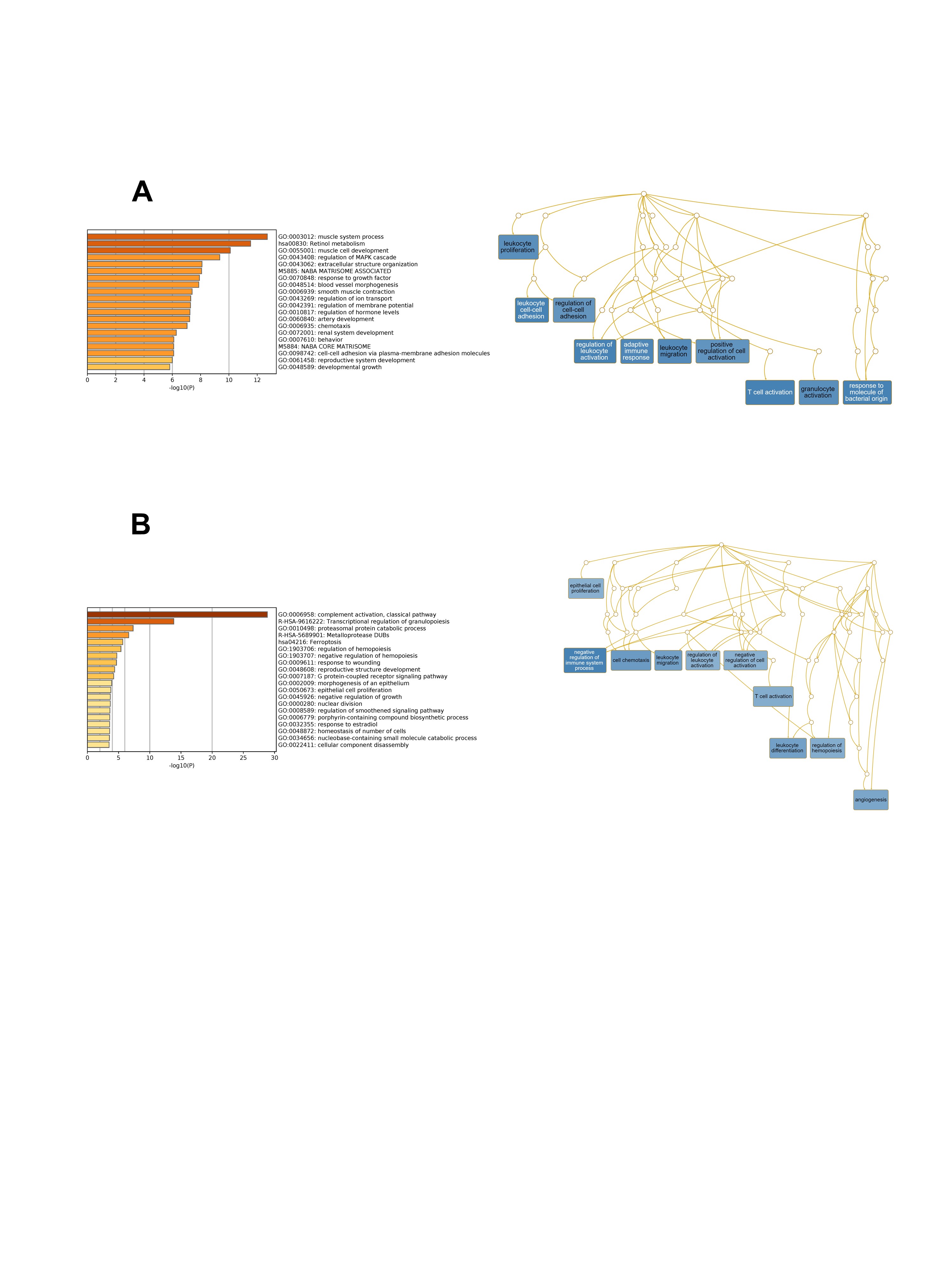

Supplement: Supplementary file 4 [file Image1.JPEG]

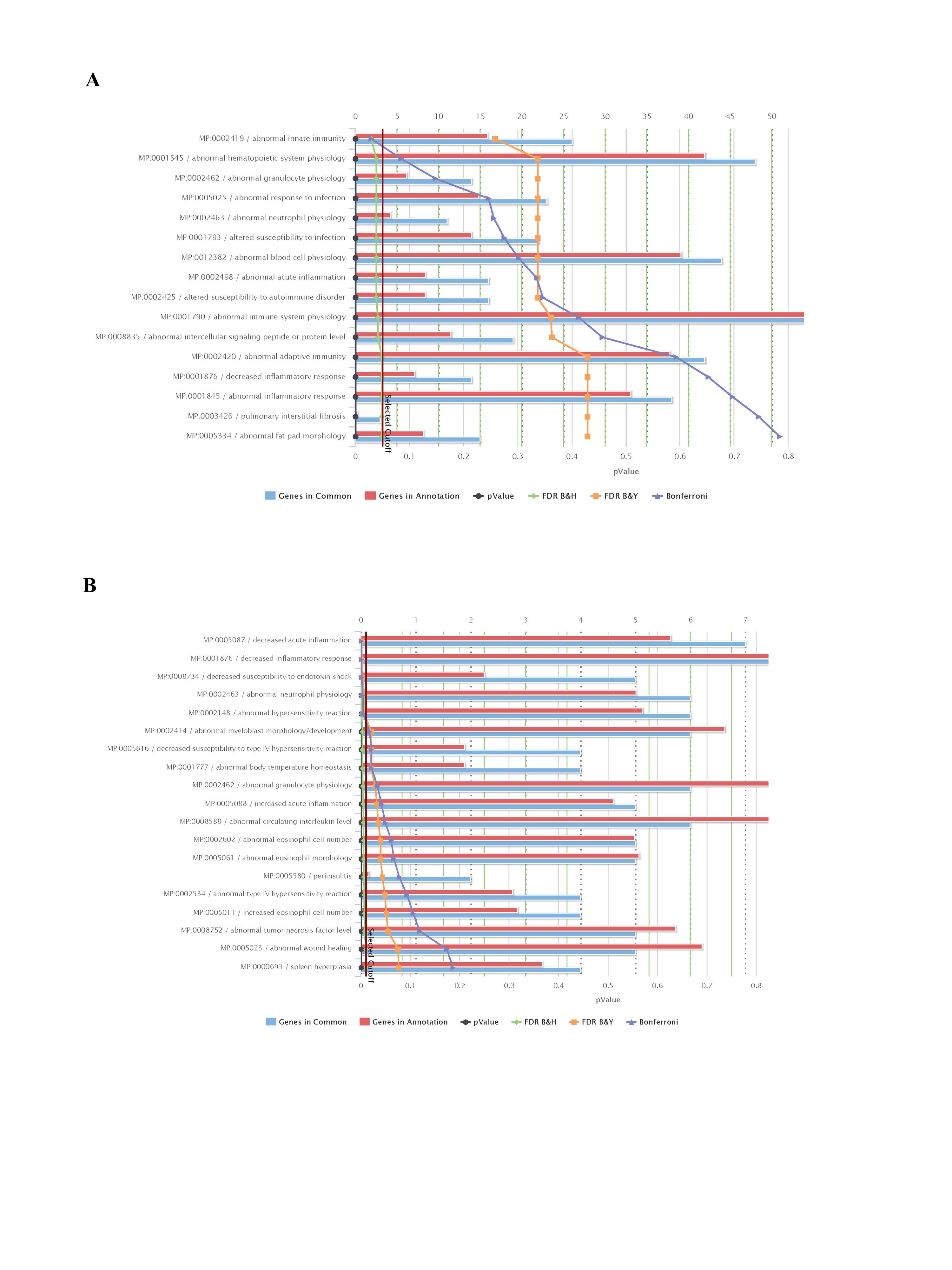

Supplement: Supplementary file 5 [file Image4.JPEG]

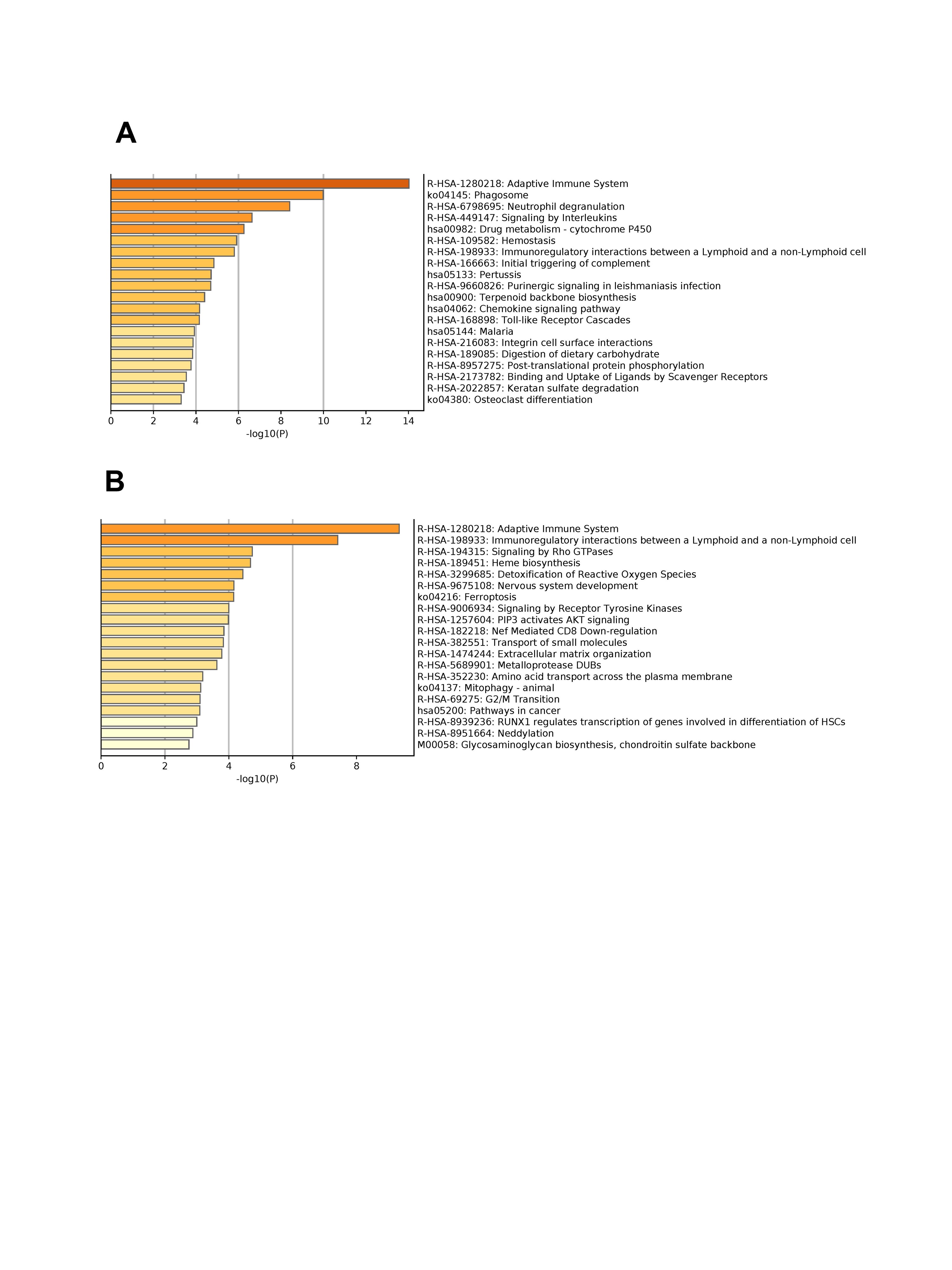

Supplement: Supplementary file 6 [file Image2.JPEG]
