# Supplementary material for: Bioinformatics insights into the genes and pathways on severe COVID-19 pathology in patients with comorbidities
Source: Front Physiol. 2022 Dec 14;13:1045469. doi: 10.3389/fphys.2022.1045469 (PMC9795193; doi:10.3389/fphys.2022.1045469)
Supplement: Supplementary file 3 [file Table1.DOCX]

|  | **Accession number** | **Sample**  **Type** | **Sample number** | **Control**  **group** | **Experimental group** | **Referances** |
| --- | --- | --- | --- | --- | --- | --- |
| **1** | **CVD** | | | | | |
|  | [GSE66360](https://www.ncbi.nlm.nih.gov/geo/query/acc.cgi?acc=GSE66360) | Endothelial Cells | 99 | 50 | 49 | (Muse et al. 2017) |
|  | [GSE83500](https://www.ncbi.nlm.nih.gov/geo/query/acc.cgi?acc=GSE83500) | Tissue | 37 | 20 | 17 | (Wongsurawat et al. 2018) |
|  | [GSE26887](https://www.ncbi.nlm.nih.gov/geo/query/acc.cgi?acc=GSE26887) | Tissue | 17 | 5 | 12 | (Greco et al. 2012) |
| **2** | **Atherosclerosis** | | | | | |
|  | [GSE43292](https://www.ncbi.nlm.nih.gov/geo/query/acc.cgi?acc=GSE43292) | Tissue | 64 | 32 | 32 | (Ayari and Bricca 2013) |
| **3** | **Diabetes** | | | | | |
|  | [GSE44314](https://www.ncbi.nlm.nih.gov/geo/query/acc.cgi?acc=GSE44314) | Blood | 11 | 6 | 5 | (Nakata et al. 2013) |
|  | [GSE55100](https://www.ncbi.nlm.nih.gov/geo/query/acc.cgi?acc=GSE55100) | Blood | 22 | 10 | 12 | (Yang et al. 2015) |
| **4** | **Obese** | | | | | |
|  | [GSE55205](https://www.ncbi.nlm.nih.gov/geo/query/acc.cgi?acc=GSE55205) | PBMC | 23 | 6 | 17 | (Jung et al. 2016) |
|  | [GSE12050](https://www.ncbi.nlm.nih.gov/geo/query/acc.cgi?acc=GSE12050) | Tissue | 18 | 9 | 9 | (Mutch et al. 2009) |
|  | [GSE9624](https://www.ncbi.nlm.nih.gov/geo/query/acc.cgi?acc=GSE9624) | Tissue | 11 | 6 | 5 | (Aguilera et al. 2015) |
| **5** | **Lung infections** | | | | | |
|  | [GSE77087](https://www.ncbi.nlm.nih.gov/geo/query/acc.cgi?acc=GSE77087) | Blood | 84 | 23 | 61 | (de Steenhuijsen Piters et al. 2016) |
|  | [GSE34205 A](https://www.ncbi.nlm.nih.gov/geo/query/acc.cgi?acc=GSE34205) | PBMC | 61 | 10 | 51 | (Ioannidis et al. 2012) |
| **6** | **Influenza** | | | | | |
|  | [GSE27131](https://www.ncbi.nlm.nih.gov/geo/query/acc.cgi?acc=GSE27131) | PBMC | 14 | 7 | 7 patients with severe pdm(H1N1) | (Berdal et al. 2011) |
|  | [GSE21802](https://www.ncbi.nlm.nih.gov/geo/query/acc.cgi?acc=GSE21802) | Blood | 23 | 3 | 9 early period | (Bermejo-Martin et al. 2010) |
|  |  |  |  |  | 12 late period |  |
|  | [GSE34205 B](https://www.ncbi.nlm.nih.gov/geo/query/acc.cgi?acc=GSE34205) | PBMC | 41 | 12 | 28 | (Ioannidis et al. 2012) |
| **7** | **HCoV-EMC** | | | | | |
|  | [GSE56677](https://www.ncbi.nlm.nih.gov/geo/query/acc.cgi?acc=GSE56677) |  | 6 | 3 | 3 | (Selinger et al. 2014) |
|  | [GSE45042](https://www.ncbi.nlm.nih.gov/geo/query/acc.cgi?acc=GSE45042) |  | 6 | 3 | 3 | (Josset et al. 2013) |
| **8** | **COVID-19** | | | | | |
|  | [GSE150316](https://www.ncbi.nlm.nih.gov/geo/query/acc.cgi?acc=GSE150316) | Tissue | 21 | 5 | 16 | (Desai et al. 2020) |
|  | [CNP0001126](https://db.cngb.org/search/project/CNP0001126/) | Blood | 35 | 64 | severe n = 22, and critical n = 13 | (Wu et al. 2020) |

**Table S1: Description of GEO datasets used in the study**

* Summary of each dataset including the GEO accession number, sample type, sample sizes, and reference to the original publication.

Aguilera, Concepción M., Carolina Gomez-Llorente, Inés Tofe, Mercedes Gil-Campos, Ramón Cañete, and Ángel Gil. 2015. “Genome-Wide Expression in Visceral Adipose Tissue from Obese Prepubertal Children.” *International Journal of Molecular Sciences* 16 (4): 7723–37. https://doi.org/10.3390/ijms16047723.

Ayari, Hanène, and Giampiero Bricca. 2013. “Identification of Two Genes Potentially Associated in Iron-Heme Homeostasis in Human Carotid Plaque Using Microarray Analysis.” *Journal of Biosciences* 38 (2): 311–15. https://doi.org/10.1007/s12038-013-9310-2.

Berdal, Jan-Erik, Tom E. Mollnes, Torgun Wæhre, Ole K. Olstad, Bente Halvorsen, Thor Ueland, Jon H. Laake, et al. 2011. “Excessive Innate Immune Response and Mutant D222G/N in Severe A (H1N1) Pandemic Influenza.” *The Journal of Infection* 63 (4): 308–16. https://doi.org/10.1016/j.jinf.2011.07.004.

Bermejo-Martin, Jesus F., Ignacio Martin-Loeches, Jordi Rello, Andres Antón, Raquel Almansa, Luoling Xu, Guillermo Lopez-Campos, et al. 2010. “Host Adaptive Immunity Deficiency in Severe Pandemic Influenza.” *Critical Care (London, England)* 14 (5): R167. https://doi.org/10.1186/cc9259.

Desai, Niyati, Azfar Neyaz, Annamaria Szabolcs, Angela R. Shih, Jonathan H. Chen, Vishal Thapar, Linda T. Nieman, et al. 2020. “Temporal and Spatial Heterogeneity of Host Response to SARS-CoV-2 Pulmonary Infection.” *Nature Communications* 11 (1): 6319. https://doi.org/10.1038/s41467-020-20139-7.

Greco, Simona, Pasquale Fasanaro, Serenella Castelvecchio, Yuri D’Alessandra, Diego Arcelli, Marisa Di Donato, Alexis Malavazos, Maurizio C. Capogrossi, Lorenzo Menicanti, and Fabio Martelli. 2012. “MicroRNA Dysregulation in Diabetic Ischemic Heart Failure Patients.” *Diabetes* 61 (6): 1633–41. https://doi.org/10.2337/db11-0952.

Ioannidis, Ioannis, Beth McNally, Meredith Willette, Mark E. Peeples, Damien Chaussabel, Joan E. Durbin, Octavio Ramilo, Asuncion Mejias, and Emilio Flaño. 2012. “Plasticity and Virus Specificity of the Airway Epithelial Cell Immune Response during Respiratory Virus Infection.” *Journal of Virology* 86 (10): 5422–36. https://doi.org/10.1128/JVI.06757-11.

Josset, Laurence, Vineet D. Menachery, Lisa E. Gralinski, Sudhakar Agnihothram, Pavel Sova, Victoria S. Carter, Boyd L. Yount, Rachel L. Graham, Ralph S. Baric, and Michael G. Katze. 2013. “Cell Host Response to Infection with Novel Human Coronavirus EMC Predicts Potential Antivirals and Important Differences with SARS Coronavirus.” *MBio* 4 (3): e00165-00113. https://doi.org/10.1128/mBio.00165-13.

Jung, Un Ju, Yu Ri Seo, Ri Ryu, and Myung-Sook Choi. 2016. “Differences in Metabolic Biomarkers in the Blood and Gene Expression Profiles of Peripheral Blood Mononuclear Cells among Normal Weight, Mildly Obese and Moderately Obese Subjects.” *British Journal of Nutrition* 116 (6): 1022–32. https://doi.org/10.1017/S0007114516002993.

Muse, Evan D., Eric R. Kramer, Haiying Wang, Paddy Barrett, Fereshteh Parviz, Mark A. Novotny, Roger S. Lasken, et al. 2017. “A Whole Blood Molecular Signature for Acute Myocardial Infarction.” *Scientific Reports* 7 (1): 12268. https://doi.org/10.1038/s41598-017-12166-0.

Mutch, David M., Joan Tordjman, Véronique Pelloux, Blaise Hanczar, Corneliu Henegar, Christine Poitou, Nicolas Veyrie, Jean-Daniel Zucker, and Karine Clément. 2009. “Needle and Surgical Biopsy Techniques Differentially Affect Adipose Tissue Gene Expression Profiles.” *The American Journal of Clinical Nutrition* 89 (1): 51–57. https://doi.org/10.3945/ajcn.2008.26802.

Nakata, Shinsuke, Akihisa Imagawa, Yugo Miyata, Atsushi Yoshikawa, Junji Kozawa, Kohei Okita, Tohru Funahashi, et al. 2013. “Low Gene Expression Levels of Activating Receptors of Natural Killer Cells (NKG2E and CD94) in Patients with Fulminant Type 1 Diabetes.” *Immunology Letters* 156 (1–2): 149–55. https://doi.org/10.1016/j.imlet.2013.10.004.

Selinger, Christian, Jennifer Tisoncik-Go, Vineet D. Menachery, Sudhakar Agnihothram, G. Lynn Law, Jean Chang, Sara M. Kelly, Pavel Sova, Ralph S. Baric, and Michael G. Katze. 2014. “Cytokine Systems Approach Demonstrates Differences in Innate and Pro-Inflammatory Host Responses between Genetically Distinct MERS-CoV Isolates.” *BMC Genomics* 15 (December): 1161. https://doi.org/10.1186/1471-2164-15-1161.

Steenhuijsen Piters, Wouter A. A. de, Santtu Heinonen, Raiza Hasrat, Eleonora Bunsow, Bennett Smith, Maria-Carmen Suarez-Arrabal, Damien Chaussabel, et al. 2016. “Nasopharyngeal Microbiota, Host Transcriptome, and Disease Severity in Children with Respiratory Syncytial Virus Infection.” *American Journal of Respiratory and Critical Care Medicine* 194 (9): 1104–15. https://doi.org/10.1164/rccm.201602-0220OC.

Wongsurawat, Thidathip, Chin Cheng Woo, Antonis Giannakakis, Xiao Yun Lin, Esther Sok Hwee Cheow, Chuen Neng Lee, Mark Richards, et al. 2018. “Distinctive Molecular Signature and Activated Signaling Pathways in Aortic Smooth Muscle Cells of Patients with Myocardial Infarction.” *Atherosclerosis* 271 (April): 237–44. https://doi.org/10.1016/j.atherosclerosis.2018.01.024.

Wu, Peng, Dongsheng Chen, Wencheng Ding, Ping Wu, Hongyan Hou, Yong Bai, Yuwen Zhou, et al. 2020. “The Trans-Omics Landscape of COVID-19.” *MedRxiv*, July, 2020.07.17.20155150. https://doi.org/10.1101/2020.07.17.20155150.

Yang, Minglan, Lei Ye, Bokai Wang, Jie Gao, Ruixin Liu, Jie Hong, Weiqing Wang, Weiqiong Gu, and Guang Ning. 2015. “Decreased MiR-146 Expression in Peripheral Blood Mononuclear Cells Is Correlated with Ongoing Islet Autoimmunity in Type 1 Diabetes Patients 1miR-146.” *Journal of Diabetes* 7 (2): 158–65. https://doi.org/10.1111/1753-0407.12163.
